# Nutritional Composition and Bioactive Compounds of Native Brazilian Fruits of the Arecaceae Family and Its Potential Applications for Health Promotion

**DOI:** 10.3390/nu14194009

**Published:** 2022-09-27

**Authors:** Rômulo Alves Morais, Gerson Lopes Teixeira, Sandra Regina Salvador Ferreira, Alejandro Cifuentes, Jane Mara Block

**Affiliations:** 1 Graduate Program in Food Science, Department of Food Science and Technology, Federal University of Santa Catarina (UFSC), Florianópolis 88034-001, Brazil; 2 Chemical and Food Engineering Department, Federal University of Santa Catarina (UFSC), Florianópolis 88040-900, Brazil; 3 Foodomics Laboratory, Institute of Food Science Research (CIAL), Spanish National Research Council (CSIC), 28049 Madrid, Spain

**Keywords:** palm fruits, phenolic compounds, biological properties, health benefit, *Euterpe edulis*, *Euterpe oleracea*, micronutrients, phytochemicals

## Abstract

The fruits from the Arecaceae family, although being rich in bioactive compounds with potential benefits to health, have been underexplored. Studies on their composition, bioactive compounds, and effects of their consumption on health are also scarce. This review presents the composition of macro- and micronutrients, and bioactive compounds of fruits of the Arecaceae family such as bacaba, patawa, juçara, açaí, buriti, buritirana, and butiá. The potential use and reported effects of its consumption on health are also presented. The knowledge of these underutilized fruits is important to encourage production, commercialization, processing, and consumption. It can also stimulate their full use and improve the economy and social condition of the population where these fruits are found. Furthermore, it may help in future research on the composition, health effects, and new product development. Arecaceae fruits presented in this review are currently used as raw materials for producing beverages, candies, jams, popsicles, ice creams, energy drinks, and edible oils. The reported studies show that they are rich in phenolic compounds, carotenoids, anthocyanins, tocopherols, minerals, vitamins, amino acids, and fatty acids. Moreover, the consumption of these compounds has been associated with anti-inflammatory, antiproliferative, antiobesity, and cardioprotective effects. These fruits have potential to be used in food, pharmaceutical, and cosmetic industries. Despite their potential, some of them, such as buritirana and butiá, have been little explored and limited research has been conducted on their composition, biological effects, and applications. Therefore, more detailed investigations on the composition and mechanism of action based on in vitro and/or in vivo studies are needed for fruits from the Arecaceae family.

## 1. Introduction

Brazilian biodiversity is known for sheltering 15% of the total number of live species in the world [1]. Although many native nuts and fruits have potential for industrial exploitation and can be an income source for small producers, most of them are still unknown and underexplored [2]. In Brazil it is possible to find 113 genera and 704 species of the Arecaceae family, which comprises approximately 704 genera and 3819 species. *Geonoma*, *Syagrus*, *Bactris*, *Atallea*, *Allagoptera*, *Astrocaryum*, and *Euterpe* are the genera with the highest number of occurrences worldwide [3]. Recently, the lipid composition [4], nutraceutical potential [5], and the chemical properties [6] of different species of fruits from the Arecaceae family have been reported. Bacaba (*Oenocarpus bacaba*), patawa (*Oenocarpus bataua*), juçara (*Euterpe edulis*), açaí (*Euterpe oleracea*), buriti (*Mauritia flexuosa*), buritirana (*Mauritiella armata* (Mart.), and butiá (*Butia odorata*) are the main fruits studied from this family due to their economic and industrial potential [7,8,9,10,11]. The production of açaí reached 1.7 million tons in 2020, which represented an income of USD 800 million in the Brazilian economy. The production of buriti in northern and northeastern regions of Brazil was 476 tons in 2020, resulting in an income of USD 471,000 [11,12]. The production of juçara fruits, which is concentrated in southern Brazil, is around 9.2 kg per plant per year. The annual productivity of 2.53 tons of fruit per hectare indicates a promising commodity [13].

**Figure 1:**
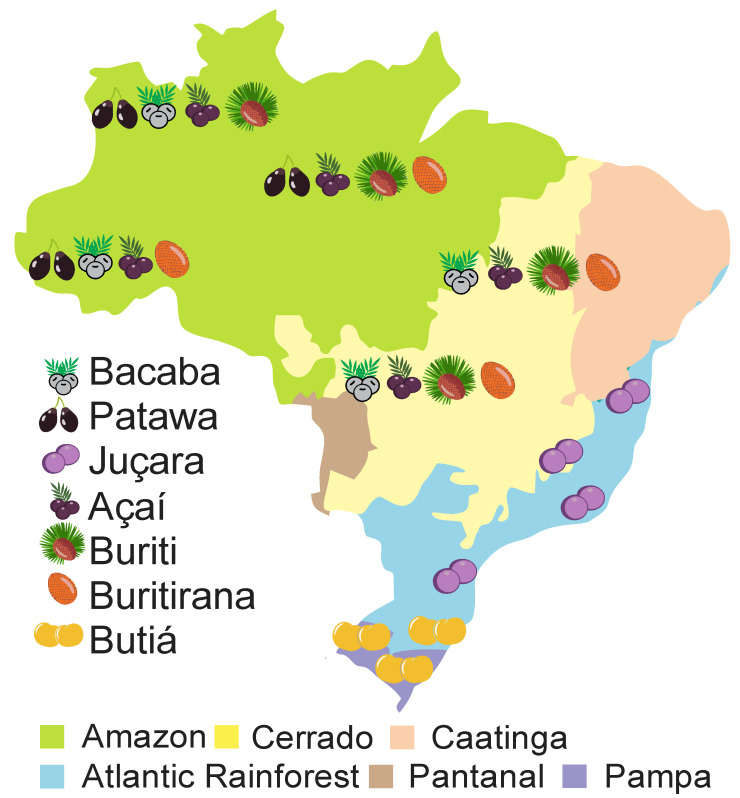
Distribution of seven native Brazilian fruits belonging to the Arecaceae family, across six biomes: Amazon, Cerrado, Atlantic Rainforest, Caatinga, Pantanal, and Pampa.

The consumption of native fruits in tropical regions has been growing due to their antioxidant, anti-inflammatory, and hypocholesterolemic activity claims [14,15,16,17]. These effects have been related to their high content of vitamins, carotenoids, and polyphenols [18,19]. These fruits are usually consumed in natura or used as ingredients to make jam, ice cream, juice, and fermented drinks. However, except for açaí, the commercialization of fruits of native Brazilian species is not very impressive. Most of them are destined for regional trade or are cultivated specifically for landscape purposes [20,21,22]. The uses, chemical and biological characteristics, and potential applications of seven native fruits of the Arecaceae family are presented and discussed in this review. The volume of production, economic potential, and studies reported in the literature were the parameters considered to choose the fruits. Studies published in Portuguese, English, and Spanish between 1990 and 2022 were selected. Nutritional composition, bioactive compounds, antioxidant activity, antimicrobial properties, and effects on health were the key words used as research target. Scopus^®^, Web of Science Core Collection, Google Scholar, Scielo, and Global Biodiversity Information Facility (GBIF) were used as research databases. The distribution of bacaba, patawa, juçara, açaí, buriti, buritirana, and butiá in the Brazilian biomes Amazon, Cerrado, Caatinga, Pantanal, Atlantic Forest, and Pampa are shown in Figure 1. The ethnobotanical characteristics, nutritional composition, minerals, lipid profile, amino acids, bioactive compounds, and the health benefits of these fruits are presented and discussed in the following sections.

## 2. Ethnobotanical Characteristics

### 2.1. Bacaba (Oenocarpus bacaba Mart.)

Bacaba, a palm native to the Amazon rainforest, is found mainly in secondary forests and in floodplains in the states of northern Brazil, such as Tocantins, Pará, and Amazonas. It is also known as bacaba verdadeira, red bacaba, bacaba-açu, bacaba-de-azeite, and bacabão. Bacaba is also present in other countries in South America where it has different names. In Peru, bacaba is named as ungurauy, in Colombia as punáma, and in French Guyana as camou [23,24].

**Figure 2:**
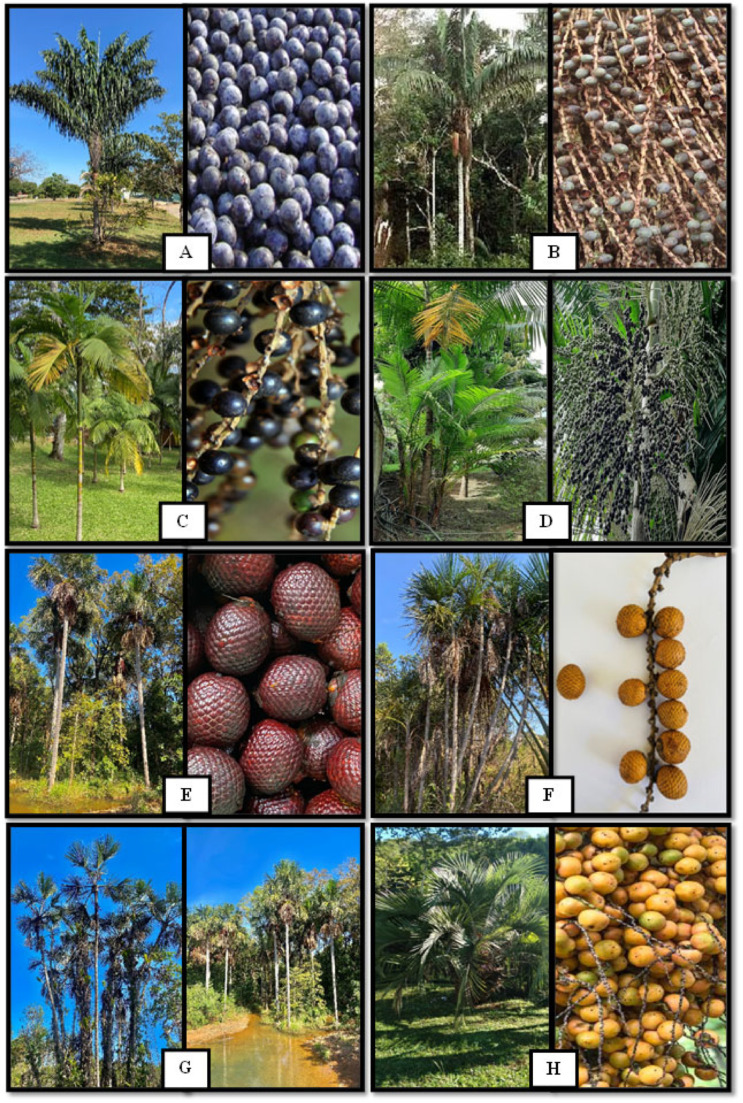
Arecaceae palm trees and their fruits: (**A**) bacaba (*Oenocarpus bacaba*), (**B**) patawa (*Oenocarpus bataua*), (**C**) juçara (*Euterpe edulis*), (**D**) açaí (*Euterpe oleracea*), (**E**) buriti (*Mauritia flexuosa*), (**F**) buritirana (*Mauritiella armata*, (**G**) similarity between buriti and buritirana, and (**H**) butiá (*Butia odorata)*. Images: Rômulo Alves Morais.

The bacaba palm tree has between 7 and 20 m of length, 15 to 25 cm of diameter, and a short thick palm heart at the apex. The pinnate leaves, usually between 8 to 17, are regularly grouped and arranged in different planes [25,26]. The bunches are robust, with rounded fruits with a dark purple color and one seed (Figure 2A) [27]. The fruiting period, which happens from January to April [23], results in a subglobose drupe from 1.4 to 2 cm in diameter and 1.5 to 3 g. The ripe epicarp has a dark purple color, while the mesocarp, which has from 1 to 1.5 mm of thickness, is lighter, oily, and fibrous (Figure 2A) [26]. The production of bacaba can reach 8 kg per plant/year [28]. The pulp of the fruit is consumed in natura, frozen, as jam, ice cream, and fermented drinks [9,25]. The palm hearts and the oil from the kernel are also consumed by the local population [29].

### 2.2. Patawa (Oenocarpus bataua Mart.)

Patawa is a palm tree native to the Amazon region (Figure 2B) [30] which grows in waterlogged soils located in lowlands and highlands (from 50 to 500 m in altitude). Patawa is popularly known as batauá, patauá (Brazil), milpesos, seje (Colombia), trupa (Colombia, Panama), komboe (Suriname), chapil (Ecuador), aricaguá (Venezuela), patawa (French Guyana), and turu (Guyana) [31]. The production of patawa in 2015 was estimated to be 11 tons of fruit/ha [32].

The stems of the patawa palm tree have up to 26 m height and diameter from 15 to 45 cm (Figure 2B). The leaves can reach up to 8 m in length, arranged regularly and in the same plane on each side of the rachis [31]. The fruits, which are smooth with an ellipsoid or ovoid format, have an average diameter of 3.5 × 1.8 cm. The color is dark purple and is usually covered by a whitish layer (Figure 2B). Patawa pulp is used to prepare a nutritive and energetic juice. The oil extracted from its fruit pulp can be used as meat preservatives and as fuel for handmade lighting in remote communities in the Amazon, where there is no electricity [30].

### 2.3. Juçara (Euterpe edulis Mart.)

Juçara, a palm tree native to the Brazilian Atlantic Forest, is found mainly between the states of Bahia and Rio Grande do Sul, and in the riparian forests in the states of Minas Gerais, Goiás, Mato Grosso do Sul, São Paulo, and Paraná. It is also found in the northeast of Argentina and the southeast of Paraguay in tropical forests between sea level and up to 1000 meters altitude (Figure 2C). It is popularly known as juçara, juçara palm, sweet palm heart, lath, içara, or palm tree [33,34].

The stem of juçara measures between 20 and 25 meters in height and has from 10 to 15 cm of diameter [33,35]. The skin is green during the period that precedes the complete maturation of the fruit, changing gradually to purple or black when the fruit is fully ripe (Figure 2C). The fruits contain a single light brown seed that represents about 90% of the fruit diameter (1–2 cm) and up to 90% of its weight (0.7–1.9 g) [36,37,38]. The fleshy mesocarp is located between the shell (epicarp) and the endocarp (seed). The immature endocarp is easily ruptured, acquiring a hard consistency when the fruit is ripe and purple. The palm heart from juçara has a shape and color similar to açaí and is known as “Açaí da Mata Atlantica”. It has been also reported that juçara fruits have nutritional composition and sensory properties similar to açaí fruits [39,40]. The fruits of juçara and açaí are not consumed fresh due to the low yield of their pulp [38,41]. They are usually processed, and the pulp is commercialized, used frozen for consumption [8] or used to make juice, jam, and ice cream.

### 2.4. Açaí (Euterpe oleracea Mart.)

The açaí is native to the Amazon where it occurs mainly in floodplains and in permanently flooded areas. On the other hand, it can also grow in dry lands in low-density forests. The moist soil and the high light intensity, due to the limited vegetation cover in lowland soils, are favorable to the development of hydrophilic vegetation species such as açaí [42,43,44]. The fruiting of açaí occurs in all seasons of the year, mainly between the months of July and December when the humidity is lower in the Amazon region [45].

The stem of açaí is smooth, thin, generally straight, and gray. It presents between 9 and 15 leaves, has height from 10 to 15 meters, and a diameter of 12 to 18 cm when the tree reaches the adult age (Figure 2D). The fruit of the açaí palm, which is composed of seed and pulp, has a globular and round shape with a diameter from 1 to 2 cm and a weight from 0.8 to 2.3 g. The fruits, when ripe, have a color ranging from dark purple to black. The white açaí produces green fruits when it is ripe [33,46,47]. The açaí pulp represents from 5 to 15% of the fruit, varying according to the origin and harvest season. The pericarp is partially fibrous and poor in starch, proteins, and lipids. The endosperm is rich in hemicelluloses, cellulose, and inulin crystals when the fruit is ripe; however, the concentration of lipids increases before the final period of maturation [45,48,49].

The açaí, which is widely consumed by the population of northern Brazil, has been reported as a superfruit. In the last decade, the açai showed a growing demand in the national and international market, attracting investments and research. As a result, the Brazilian production of processed açaí pulp grew about 89% between 2010 and 2020, reaching approximately 1.7 million tons and generating about USD 800 million for the Brazilian economy in 2020 [11,50]. The processing of açai needs to be performed up to 12 hours after the harvesting, since the fruit degrades rapidly under the high temperatures observed in the north and northeast of Brazil [33]. The açaí is used as an ingredient in yogurts, candies, juices, nectars, and jam.

### 2.5. Buriti (Mauritia flexuosa L.f.)

Buriti is a palm tree found mainly in the biomes Amazon and Cerrado and it is also known as miriti, buriti coconut, buriti palm, and swamp palm. It grows in swamps close to permanent watercourses and on top of mountains, which is an advantage since these areas are not suitable for other activities [51]. The buriti palm tree usually reaches more than 15 meters and specimens have been reported reaching more than 50 meters. The leaves have a fan-like shape (Figure 2F) [51,52].

The fructification of the buriti happens between December and June, and each plant can produce between 150 and 200 kg of fruit per harvest. The fruits have an average weight of 50 g, longitudinal diameter of 5.25 cm, and a cross-sectional diameter of 3.91 cm (Figure 2E) [53,54]. The shape is elliptical oval, surrounded by a pericarp (shell) composed of triangular scales of dark and hard red color. The mesocarp (pulp) is thin and soft, with a bittersweet flavor, striking and peculiar aroma, and dark red to yellow color. The fibers are used to make ropes and hammocks and leaf petioles to make bottle stoppers, toys, rustic beds, and rafts [55,56]. The pulp of buriti is used by the local population for preparing juice, marmalades, jams, ice cream, wine, and fermented beverages. The foodstuffs and beverages made with the fruit are also sold in local markets, generating income for the population and ensuring the maintenance of the local culture [57]. In addition, its oil and pulp are commonly used to prevent and treat some pathologies due to their potential antimutagenic, antibacterial, and healing properties [10,58].

### 2.6. Buritirana (Mauritiella armata Mart.)

Buritirana is also known as buriti mirim, buriti bravo, caranã in Brazil, and aguajillo in Colombia and Venezuela. The fruits are globose to oblong–ellipsoid (Figure 2F). The pulp is fleshy and fibrous with a slightly reddish color and a strong and peculiar aroma. The endocarp is very thin and surrounds a hard seed. The shell, which is similar to the buriti shell, presents overlapping scales and a reddish-brown color. Its consumption by local populations is mainly in natura and it is also used to make drinks, wines, and sweets [6,59,60].

Figure 2G shows that the buriti and buritirana palm have similar fruits and leaves. The difference between these species is the stem. The buriti has a single stem with a diameter of 45 cm. On the other hand, the buritirana presents a stem divided in several segments with a diameter of 20 cm. The buritirana has globose to oblong fruits, fleshy and fibrous pulp, and a very thin endocarp surrounding a hard seed. It is consumed by local populations in natura or used in beverages, sweets, and wines [59,60,61].

### 2.7. Butiá (Butia odorata (Barb. Rodr.) Noblick)

The *Butia odorata* is a palm tree which is native to southern Brazil and east of Uruguay. It usually grows in open areas such as fields, savannas, dunes, and sandbanks in the Pampa biome [62], and is usually found in flat and flooded terrain, flowering between September and January. The peak of fruiting occurs between December and April [63]. Currently, the species in Brazil and Uruguay are considered of great vulnerability since the adult plants are centenary and suffer with the increase in the area for livestock and intensive agriculture. Human action in the native areas causes a great impact on the regeneration cycle of the trees [64,65,66].

The butiá palm tree has a single, straight, and inclined stem 3–6 m high, without visible palm hearts at the top. Its leaves (7–32) are pinnate, grey-green, and serrated, and the fruits are pale yellow to reddish-orange, with an average diameter of 1.7 to 4.2 cm [67]. The mesocarp is fleshy, with an endocarp containing one to three locules with three pores (Figure 2H) [31,68]. The maturation of the fruits occurs mainly in summer between February and April, with maximum production in February [69]. Butiá has an intense aroma and flavor and high acidity. It is consumed fresh or used in juices, alcoholic beverages, and frozen products [70,71]. The pulp and leaves are also used to treat skin diseases and infections [55]. The commercialization of butiá can bring economic and social benefits without environmental degradation. Therefore, it is interesting to stimulate its research and sustainable production.

## 3. Macro and Micronutrients

Table 1 shows the macro- and micronutrient composition for fruits of the Arecaceae family. The moisture (from 30.36 to 88.90 g 100 g^−1^), lipids (2.18 to 21.02 g 100 g^−1^), and energy values (64.68 to 368.78 kcal 100 g^−1^) show great variation. The pulps of bacaba and buritirana have the highest content of lipids and energy value (21.02 and 21.01 g 100 g^−1^; 377.54 and 368.78 kcal 100 g^−1^, respectively). On the other hand, the butiá pulp shows the lowest values for these parameters (2.18 g 100 g^−1^ and 70.46 kcal 100 g^−1^, respectively). The buritirana and açaí pulp show the highest protein content (5.96 and 5.30 g 100 g^−1^, respectively). Açaí, patawa, and bacaba present the highest content for carbohydrates (47.83, 46.10, and 42.80 g 100 g^−1^, respectively), and the lowest value is observed for juçara palm (5.46 g 100 g^−1^). The highest fiber content was reported for buritirana (65.46 g 100 g ^1^), followed by patawa pulp (29.70 g 100 g^−1^). These fruits are richer in fibers when compared to commercial and popular fruits such as banana (10.50 g 100 g^−1^), mango (6.71 g 100 g^−1^), watermelon (8.73 g 100 g^−1^), and tamarind (13.93 g 100 g^−1^) [72]. The ingestion of 100 g of buritirana pulp can supply the recommended dietary intake (RDI) of fiber for healthy adults, which is 25–35 g on a 2000 kcal diet [73]. The patawa pulp, which represents 40% of the fruit weight, is rich in proteins (4.90%), oil (14.40%), and carbohydrates (46.10%) [74]. Butiá and buriti showed lower levels of fiber (1.31 and 6.02 g 100 g^−1^, respectively). A diet rich in fiber has been related with health benefits such as blood pressure reduction, improvement in serum lipid profile, and glycemic control [73,75].

The consumption of minerals is necessary for the proper functioning of the organism, and they are related to energy at the cellular level and macronutrient metabolism [76]. Moreover, the minerals are part of molecules such as vitamins, amino acids, hormones, and blood cells. Ca, Mg, and K are needed in higher amounts and Zn, Cu, I, Mn, and Se in lower levels [77]. The main mineral found in açaí, buritirana, and butiá was potassium (K) (930.00, 672.25, and 462.40 mg 100 g^−1^, respectively). The RDI of K, which contributes to the reduction in blood pressure and the risk of cardiovascular diseases, is 3510 mg per day for healthy adults [78]. The consumption of 100 g of açaí, buritirana, and butiá fruit pulp represent 26.48%, 19.15%, and 13.23%, respectively, of the RDI of K, which is 3510 mg per day for a healthy adult. A deficiency of K in the diet can result in fatigue, leg cramps, muscle weakness, slow reflexes, acne, dry skin, and irregular heartbeat, among other symptoms [79,80].

Juçara, açaí, buritirana, and buriti are rich in Ca (up to 462 mg 100 g^−1^) and Mg (up to 317 mg 100 g^−1^). Considering the RDI for a healthy adult for Ca (1300 mg per day) and Mg (400 mg per day), the intake of 100 g of açaí represents 36 and 76% of the RDI for Ca and Mg, respectively. Magnesium and calcium form stable complexes with phospholipids that are part of cell membranes. The action of these minerals, which can act synergistically, depends on its the concentration in the cells [94]. Other minerals present in the fruits of the Arecaceae family in intermediate concentrations are Na (1.90–71.21 mg 100 g^−1^), Mn (0.61–45 mg 100 g^−1^), I (0.28–46.60 mg 100 g^−1^), and P (6.90–186 mg 100 g^−1^). Moreover, the highest concentrations of these minerals were found in the same fruits with higher potassium levels. Schulz et al. [40] observed the same behavior in dark-colored fruits found in Brazil, such as *Myrcianthes pungens, Myrciaria cauliflora,* and *E. edulis.* The consumption of 100 g of juçara and açaí provides more than 100% of the RDI for Mn (2.3 mg day^−1^) and iron (8 mg day^−1^). Furthermore, 100 g of buritirana can afford more than 100% of Mn, and 36% of I. 

The buriti pulp showed a high level of Se (0.05 mg 100 g^−1^) and Cr (0.12 mg 100 g^−1^). The RDI for these minerals is 0.055 mg per day for Se and 0.03 mg per day for Cr. Chromium is linked to gene expression, energy production, synthesis of lipoproteins or lipids, and regulation of glucose metabolism [95]. The deficiency of Cr in the human organism can cause glucose intolerance, weight loss, peripheral neuropathy, and increase for the risk of cardiovascular disease [96]. The selenium is related to the function of the thyroid and immune system. It is associated with a reduction in the risks of several types of cancer [97], and its deficiency can contribute to cardiovascular disease, hypothyroidism, and deficiencies of the immune system [95].

### 3.1. Lipid Profile

Table 2 shows the lipid profile of the fruits from the Arecaceae family. The main fatty acids reported in these fruits were oleic (C18:1) > palmitic (C16:0) > linoleic (C18:2) > stearic (C18:0) > linolenic (C18:3). A content of 75.7, 72.7, and 52.1% of oleic acid was reported for the pulp of buriti [74], patawa [98], and açaí [99], respectively. The consumption of oils with a high content of oleic acid has been associated with the reduction of cholesterol. This fatty acid also presents higher oxidative stability when compared to polyunsaturated fatty acids (PUFAs) [98].

The linoleic acid, an essential PUFA, was found in açaí (48.05%), butiá (32.80%), and juçara (26.10%). The majority of the fruits present a balanced fatty acid composition, with high content in monounsaturated fatty acids (MUFAs) and saturated fatty acids (SFAs). In addition, the high levels of unsaturated fatty acids in its pulp make this raw material susceptible to oxidation reactions which may cause physical and sensory changes [100]. The patawa presented the lowest concentration of PUFA (2.72%). It has been reported that the fruit patawa has potential for the production of edible oil suitable as an ingredient in cosmetics, soaps, and foods such as popsicles, ice cream, and concentrated juices [74,98]. Its oil still has antimicrobial activity and high oxidative stability compared to other commercial oils [4,7]. Despite its interesting nutritional composition, patawa is still relatively unknown in Brazil, and it is used only by the local population in the regions where it is grown [101]. On the other hand, buriti oil has a lipid content of 22%, composed mainly of oleic acid (72.23%) and palmitic acid (21.18%). The nutritional composition of the fruits depends on the place of cultivation, soil, and genotype. The buriti oil is rich in unsaturated fatty acids and carotenoids. Oliveira et al. [102] reported an antioxidant and antidiabetic effect at low concentrations of buriti oil (10 and 15 mg mL^−1^).

The oils obtained from bacaba and patawa pulp have high concentrations of SFAs (38.98 and 36.65%, respectively), mainly lauric and stearic acids. These oils can be used in the oleochemical industry and for the development of new lipid-based formulations with diverse industrial applications [103,104]. Caproic (C6:0), caprylic (C8:0), and capric (C10:0) acids are also found only in patawa oil in concentrations of 0.40, 7.80, and 8.00%, respectively.

The highest concentration of tocopherols was observed for buriti (1688.58 mg kg^−1^), followed by juçara, açaí, and patawa (1193.00, 645.00, and 341.00 mg kg ^1^, respectively). The α-tocopherol was the only tocopherol identified in açaí oil, which presented the highest concentration reported for this isomer (645.00 mg kg^−1^) [110]. This concentration is higher than those reported for extra virgin olive oil (163.00 mg kg^−1^) and other refined oils such as soybean (352.00 mg kg^−1^), sunflower (575.00 mg kg ^1^), and corn (207.00 mg kg^−1^) [116,117]. The main tocopherols identified in the butiá and buritirana were α-tocopherol, β-tocopherol, and γ-tocopherol. The α-tocopherol is the isomer with vitamin E activity, and it has been reported that it is associated with the prevention of atherosclerosis and atherosclerosis and steatosis [118].

The patawa oil showed the highest content of phytosterols, followed by bacaba and buriti (1169.10, 106,00, and 100 mg kg^−1^, respectively). The main phytosterol present was β-sitosterol. Data on the profile of the phytosterols for juçara, açaí, buritirana, and butiá were not found in the literature. It has been reported that the phytosterols can reduce the serum levels of fat-soluble vitamin E (α-tocopherols) and β-carotene, which has pro-vitamin A activity [119]. The highest concentrations of β-sitosterol were found in bacaba, patawa, and buriti (76.40, 479.20, and 76.6, respectively. It has been reported that the consumption of these fruits decreases blood cholesterol levels in hyper- and normocholesterolemic individuals [120]. Dumolt and Rideout [121] and Jones et al. [122] reported that phytosterols are considered GRAS (generally recognized as safe) and have not been correlated with any mutagenic activity or toxicity in experimental studies.

### 3.2. Amino Acids

Table 3 shows that patawa can be considered a source of essential amino acid (502.00 mg g ^1^ protein), when compared to açaí and buriti. The most prevalent amino acids in patawa were leucine, threonine, isoleucine, lysine, and valine. These branched-chain amino acids are related to the protein synthesis, as they stabilize the protein structure through hydrophobic interactions [123]. Data on the amino acid composition of bacaba, juçara, butiá, and buritirana have not been found in the literature.

Buriti is rich in threonine, leucine, and tryptophan (85.50, 23.80, and 23.80 mg g^−1^ protein, respectively). Tryptophan, a precursor of serotonin which is involved in the modulation and regulation of anxiety and mood, is not usually found in foods [124]. Tryptophan is also a precursor of compounds associated with sleep regulation and stress reduction, such as nicotinamide (vitamin B6), tryptamine, melatonin, kynurenine, xanthurenic, and quinolinic acids [125].

## 4. Bioactive Compounds

**Figure 3:**
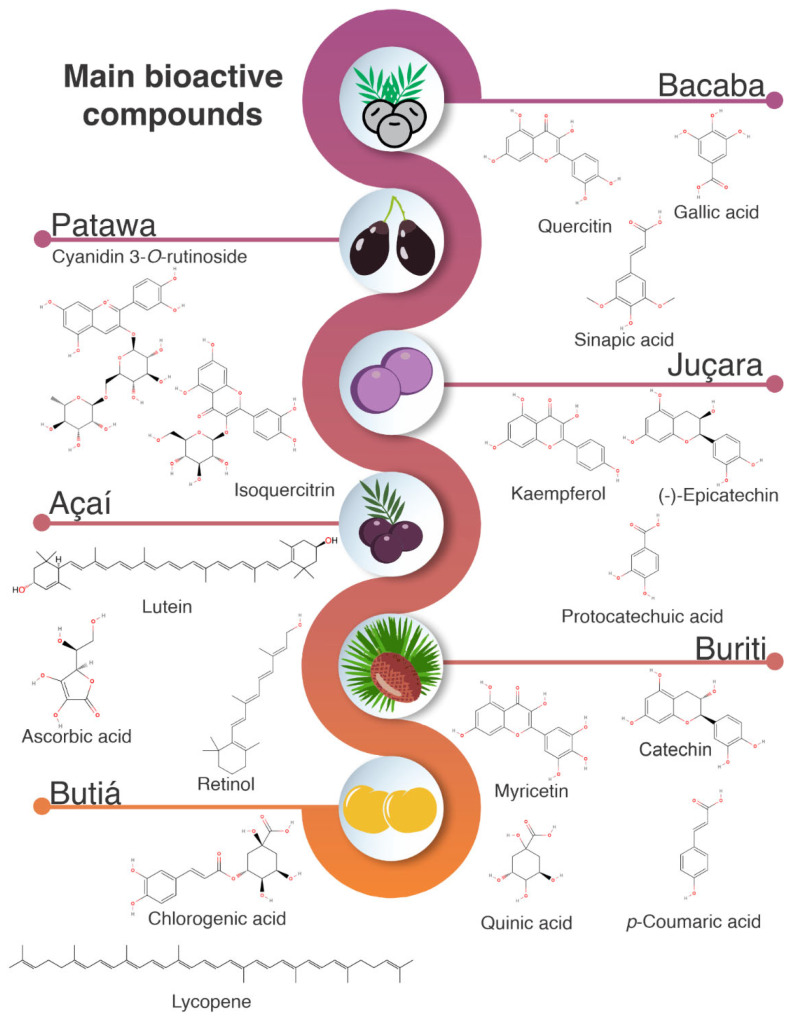
Chemical structures of the main bioactive compounds found in fruits of the Arecaceae family.

Figure 3 shows the bioactive compounds reported in bacaba, patawa juçara, açaí, buriti, buritirana, and butiá. Phenolic acids, flavonoids, anthocyanins, and vitamins were the main compounds found in these fruits. Gallic acid, cyn 3-*O*-rutinoside, rutin, and (+)-catechin in bacaba, and cyn 3-*O*-rutinoside and (−)-epicatechin in patawa were the main compounds assessed. Açaí and juçara have been reported as superfruits, and compounds such as apigenin, cyn 3-*O*-rutinoside, rutin, myricetin, quercetin, kaempferol, and vanillic acid have been reported in their composition. On the other hand, protocatechuic acid, rutin, chlorogenic acid, (−)-epicatechin, and luteolin are the main compounds found in buriti. For butiá, the main compounds are chlorogenic acid, quercetin, and myricetin. The presence of these compounds in the fruits from the Arecaceae family are presented and discussed in the next sections.

### 4.1. Phenolic Compounds

Table 4 shows the phenolic composition of the fruits of the Arecaceae family. The concentration of the total phenolic compounds (TPC) is directly proportional to the antioxidant activity (AA) in these fruits. The highest content of phenolic compounds was observed for juçara, açaí, bacaba, and butiá (5672.0, 3437.4, 1759.27, and 1250.30 mg GAE 100 g^−1^, respectively). Santos et al. (2015) reported for buriti pulp from the Amazon 118 ± 2 mg GAE 100 g^−1^ of TPC. On the other hand, Candido et al. (2015) reported that buriti pulp from Cerrado biome had higher values of phenolic compounds when compared to the fruit of the Amazon biome. Royo et al. [130] reported that buritirana has a flavonoid content of 7.92, 5.93, and 0.93 mg g^−1^ in the leaves, roots, and petioles, respectively. It has been also reported that the fruit is rich in carotenoids such as trans-β-carotene (373.00 μg 100 g^−1^), all-trans-α-carotene (230.00 μg 100 g^−1^), trans-lutein (198.00 μg 100 g^−1^) and 9-cis-β-carotene (11.00 μg 100 g^−1^) [60].

### 4.2. Anthocyanins

Açaí and juçara are rich in bioactive compounds such as cyanidin 3-glycoside and cyanidin 3-rutinoside, lutein, α-carotene, and β-carotene, which are associated with the prevention of inflammatory diseases and cancer [41,157]. A high concentration of anthocyanidins has been reported for juçara and açaí (409.85 and 110.10 mg cyanidin 3-*O*-glucoside 100g^−1^, respectively) (Table 4). This can explain the higher antioxidant activity reported for juçara when compared to buriti and bacaba, which have lower concentrations of TPC and anthocyanidins (3.10 and 34.69 mg cyanidin 3-*O*-glucoside 100 g^−1^, respectively) [158]. The main phenolic compounds reported for buriti are rutin (1460 µg 100 g^−1^), (+)-catechin (961.21 μg g^−1^), (−)-epicatechin (1109.93 μg g^−1^), and luteolin (1060.90 μg g^−1^) [152] (Figure 3). It has been reported that the high antioxidant capacity for these compounds is related to the neutralization of free radicals and chelating of transition metals [159,160].

### 4.3. Carotenoids and Vitamin C

Buriti has been reported as the richest source of carotenoids found in Brazil [153,161,162]. Lima et al. [163] reported values for β- and α-carotene of 34.085 μg 100 g^−1^ and 3.625 μg 100 g^−1^, respectively. Rosso and Mercadante [150] reported a total carotenoid content of 513.72 µg g^−1^ for buriti pulp which represents a retinol activity equivalent (RAE) of 7280 RAE 100 g^−1^. Buriti presents a higher total carotenoid content and RAE when compared to fruits considered rich in this bioactive compound such as dendê (129.03 µg g^−1^/1535 μRAE 100 g^−1^), pupunha (197.66 µg g^−1^/1491 μRAE 100 g^−1^), physalis (80.89 µg g^−1^/1108 μRAE 100 g^−1^), tucumã (62.65 µg g^−1^/850 μRAE 100 g^−1^), mamey (62.53 µg g^−1^/ 688 μRAE 100 g^−1^), and marimari (37.98 µg g^−1^/ 605 μRAE 100 g^−1^) [150]. A concentration of β- carotene of 483 μg g^−1^ (300 μRAE 100 g ^1^) has been reported for açaí. This value is higher when compared to the concentrations reported for tropical fruits such as cajá (120 μRAE 100 g^−1^) [164], mango cultivars Tommy Atkins and Keitt (96 and 251 μRAE 100 g^−1^, respectively) [165], and acerola cultivar Olivier (148–283 μRAE 100 g^−1^) [150].

Lutein, a dehydroxylated carotenoid belonging to the class of yellow-colored xanthophylls, is predominant in açaí (483 μg g^−1^) and buriti (226 μg g^−1^). The values of lutein in these fruits are higher than in vegetables considered rich in lutein, such as caruru (119 μg g^−1^), mentruz (111 μg g^−1^), and taioba (104 μg g^−1^). Moreover, the concentration of lutein in açaí is higher than the concentration reported in nasturtium (450 μg g^−1^), an edible flower that was considered the richest source of lutein reported in the literature [166]. Açaí has been reported as a super fruit [167], with carcinogenic [157] and neuroprotective [43] effects associated with its high content of lutein.

Vitamin C is the most important water-soluble antioxidant, acting on scavenging free radicals such as hydroxyl radical, superoxide, singlet oxygen, and hydrogen peroxide [168]. Acerola and camu camu are fruits rich in vitamin C (1357 and 1882 and mg 100 g^−1^, respectively) [137]. Butiá (503.4 mg 100 g^−1^) and Juçara (186 mg 100 g^−1^) have the highest content of vitamin C. Butiá presents higher amount of vitamin C when compared with mangaba (190 mg 100 g^−1^), cajá (26.5 mg 100 g^−1^), murici (148 mg 100 g^−1^), jaboticaba (238 mg 100 g^−1^) [137], pear orange (62.50 mg AA 100 mL^−1^ of juice), and Christmas orange (84.03 mg AA 100 mL^−1^ of juice) [169].

## 5. Biological Effects and Potential Health Benefits of Phenolic Compounds

### 5.1. Antioxidant Activity

The in vitro antioxidant activity (AA) observed through 2,2-diphenyl-1-picrylhydrazyl radical scavenging capacity (DPPH) and oxygen radical absorption capacity (ORAC) has been reported for the pulp of butiá [15]. The high antioxidant activity was related mainly to (+) catechin (259.18 μg g^−1^), (−)-epicatechin (211.12 μg g^−1^), and rutin (161.20 μg g^−1^). The authors also reported lower concentration of *trans*-resveratrol, sinapic and ellagic acids, apigenin, and naringenin in the pulp of butiá. These bioactive compounds are related to anticarcinogenic, cytoprotective, antioxidant, cardioprotective, and neuroprotective activity [170]. The phenolic compounds can protect cellular macromolecules from damage induced by reactive oxygen species (ROS) and reactive nitrogen species (RNS). They can regenerate biomolecules such as proteins, and lipids are highly oxidized, avoiding damaged cell death and preventing chronic diseases [171,172].

Nonato et al. [18] reported in vitro AA for buriti extracts using the ability to scavenge 2,2′-azinobis-3-ethylbenzothiazoline-6-sulfonic acid (ABTS) radicals and ferric reducing antioxidant power (FRAP). The 80.90% of chelating activity by FRAP observed for buriti was independent of the concentration (14–700 µg mL^−1^) for extracts recovered using ethyl acetate as solvent. The AA for the extracts of buriti was higher when compared to the results reported for extracts of açaí and juçara for DPPH, FRAP, and ORAC (Table 4). Buriti showed a potential to reduce the DPPH radical that was 20 times higher when compared to butiá and approximately 4 times higher when compared to açaí.

The AA using the ABTS assay was higher for patawa and açaí (2471.50 and 1154.43 μmol TE g^−1^, respectively). Considering the patawa, the authors indicated that patawa presented AA that was about 41 times higher using ABTS radical when compared to bacaba, juçara, and buriti. Considering that the ABTS radical is soluble in organic and aqueous solvents, this indicates that patawa has antioxidant components with different solubility. In addition, the absorption at a wavelength of 734 nm of the ABTS radical eliminates possible interference from color, resulting from unsatisfactory extraction processes [173]. Rezaire et al. [101] and Saravia et al. [82] reported a high content of phenolic compounds in the patawa pulp extracts with high antioxidant activity, and that patawa has higher antioxidant activity (TEAC and FRAP assays) compared to açaí. The authors indicated that patawa could be used as an ingredient in foodstuffs, cosmetics, and pharmaceutics. Medicinal properties have been reported for its pulp, leaves, and roots, such as treating hair loss, dandruff, bronchitis, tuberculosis, and malaria [174].

Buriti showed the highest antioxidant capacity in FRAP and ORAC assays (8890.00 μmol FeSO_4_ g^−1^ and 2470.00 μmol TE g^−1^, respectively), followed by patawa (1869.90 μmol FeSO_4_ g^−1^ and 1626.70 μmol TE g^−1^) and juçara (1745.33 μmol FeSO_4_ g^−1^ and 1266.36 μmol TE g^−1^). The differences observed between the AA assays are related to the differences within the assays, such as the radicals, pH, temperature, time, solvents, and method of extraction [40,175,176]. Buriti showed approximately 134 times more power to reduce the ferric ion by the FRAP and 13 times more power of reduction by the ORAC test when compared to bacaba. These higher FRAP and ORAC values observed for these fruits are probably correlated with the polyphenol contents.

Most of the in vivo studies that evaluated the AA were carried out with animals exposed to adverse conditions, such as high-fat diets, oxidative stress, and diabetes. Copetti et al. [14] evaluated the effect of acute consumption of juçara juice on the reduction of biomarkers of oxidative stress and fatigue in 15 healthy men using a HIIT protocol (high-intensity interval training). The results showed that the acute consumption of juçara juice immediately decreased the oxidative stress index (OSI) and fatigue. On the other hand, the consumption of juçara increased the levels of reduced glutathione (GSH) after 1 hour under HIIT. Moreover, the consumption of juçara juice significantly increased the content of uric acid and total phenols over time, indicating that it can induce antioxidant responses and reduce fatigue after training. It also suggests that more benefits to the human health can be achieved when practicing sports together with the consumption of juçara juice than practicing the sports only.

The AA for the pulp, shell, and seed of buriti was evaluated using thiobarbituric acid reactive substances (TBARS) and the oxidative hemolysis inhibition, DPPH, ABTS, and FRAP assays. The shell of buriti showed significantly higher AA when compared to the seed and pulp. Moreover, the bioaccessibility of phenolic compounds for pulp, shell, and seed after simulated in vitro digestion decreased from 553, 1288, and 597 mg L^−1^ to 102, 498, and 133 mg L^−1^, respectively, which represent 18.70, 38.70, and 22.30% reduction on the content of bioactive compounds. The ability of the extracts of buriti to cause hemolysis in red blood cells was determined, and even at the highest concentration (8.0 mg mL^−1^), the induction of the lysis was not observed. The authors also report that the blood cells treated with the extracts (0.5, 1.0, 2.0, 4.0, and 8.0 mg mL^−1^) were protected when exposed to the peroxide radicals produced by the thermal decomposition of AAPH (2,2′-Azobis(2-amidinopropane) dihydrochloride)) [177]. Table 5 shows in vitro and in vivo studies on antioxidant activity, anti-inflammatory, chemopreventive, cardioprotective, and antimicrobial effects of the fruits belonging to the Arecaceae family.

### 5.2. Antimicrobial Effects

The antimicrobial potential of extracts obtained from butiá has been reported [188,189,190]. Maia et al. [190] reported that the hexane extract of butiá (3 and 7 mg mL^−1^) presented activity against Gram-negative bacteria such as *Salmonella Typhimurium*, *Escherichia coli* O157: H7, and *Pseudomonas aeruginosa*), with zones of inhibition ranging from 59.50 to 86.00 mm using the agar diffusion method.

On the other hand, *Listeria monocytogenes* and *Bacilus cereus* were not inhibited at the highest concentrations tested (11 mg mL^−1^). Gram-positive bacteria are more sensitive to antimicrobial compounds from plant origin when compared to Gram-negative bacteria [192]. Such behavior may be correlated to the cellular membrane of Gram-negative bacteria, which is composed of lipopolysaccharides ensuring protection against various agents. It has been reported that (+)-catechin, (−)-epicatechin, quercetin, and phytosterols present synergistic action against the Gram-negative bacterium [188,189,193].

The extracts of butiá obtained with acetone showed activity against *Escherichia coli* inoculated in sliced cheese with a minimal inhibitory concentration (MIC) value of 15 mg mL^−1^. The logarithmic decrease observed for the number of colony forming units (CFUs) of *Escherichia coli* in the samples treated with the extract after 72 h was eight times higher when compared to the control (2.8 log CFU cm^−2^ and 0.5 log CFU cm^−2^, respectively). The authors associated the antimicrobial activity of the extract with the main compounds such as Z-10-pentadecenol (80.1%) and palmitic acid (19.4%), identified in the butiá extract [189].

Haubert et al. [188] reported that methanolic extracts of *Butiá odorata* showed antimicrobial and antibiofilm activity against 26 serovars of *Salmonella* spp. isolated from food and environment where food was prepared. The MIC of *Butiá odorata* extract ranged from 10 to 19 mg mL^−1^. This inhibition may be correlated with the presence of bioactive compounds in the extract such as phenolics, phenols, and flavonoids. The main compounds identified in the extracts were the 5-(hydroxymethyl)-2-furfural (65.17%), followed by the pyranone, 2,3-dihydro-3,5-dihydroxy-6-methyl-4H-pyran-4-one (8.49%). Polovková and Šimko [194] reported that the formation of 5-(hydroxymethyl)-2-furfural is due to the Maillard reaction or the dehydration of reducing saccharides caused by exposure to high temperatures. In this study, in the process of elaboration and characterization of the extracts, the temperatures did not exceed 40 °C. The activity of piranones against *Salmonella* spp. and Gram-negative and Gram-positive bacteria has been reported [188].

The antimicrobial activity of nonencapsulated buriti oil against *Klebsiella pneumonia*, *Pseudomonas aeruginosa*, and *Staphylococcus aureus* has been reported (Table 5). The nonencapsulated buriti oil increased the antimicrobial activity against *Pseudomonas aeruginosa* (59%), *Klebsiella pneumonia* (62%), and *Staphylococcus aureus* (43%) when compared to the control group without treatment. The inhibition of the bacterial growth was related to the particle size and the phytochemicals, such as quercetin, eugenol, and vanillic acid, present in the oil. Leão et al. [195] reported that nanoemulsions of interesterified and noninteresterified buriti oil were effective against Gram-negative bacteria.

### 5.3. Anti-Inflammatory and Hypocholesterolemic Effect

The inflammatory process is a complex immune response of the organism to heal infections or repair damaged tissue. However, inflammation can produce an uncontrolled response or can be related to the disruption of the homeostasis state of physiological processes. It can lead to chronic systemic damage and inflammatory diseases such as diabetes, asthma, Alzheimer’s, atherosclerosis, cancer, neurodegenerative, and neurological diseases [196]. The free radicals produced by active inflammatory leukocytes in chronic and acute inflammation are highly deleterious [197]. High levels of proinflammatory molecules such as C-reactive protein, nitric oxide (NO), reactive oxygen species (ROS), cyclooxygenase-2 (COX-2), tumor necrosis-α (TNF-α), interleukins (IL-1β, IL-6, IL-8), and transforming growth factor-β are found in the inflammatory process [198,199,200].

Several plants have been used in folk medicine as an alternative to the treatment of chronic inflammation with fewer side effects and low toxicity. Nonsteroidal anti-inflammatories (NSAIDs), which are the traditional anti-inflammatory drugs used, can cause adverse effects such as a decrease in COX-prostaglandin production, gastrointestinal disorders, kidney problems, and severe peptic ulcer. A positive correlation has been reported between ingestion of foods rich in phenolic compounds and a negative modulation of the inflammatory response [198,199]. The mechanism for the anti-inflammatory activity has not been elucidated yet [201].

Silva et al. [181] studied the anti-inflammatory activity of lyophilized juçara pulp in obese Wistar rats in a proinflammatory state. The animals were fed for 16 weeks with hypercaloric and hyperlipidemic diets with 0.5% and 2% of lyophilized juçara pulp, and the tumor necrosis factor-alpha (TNF-α), interleukin 1β, concentrations of lipopolysaccharides (LPS), and toll-like receptor-4 (TLR-4), which is an encoded protein, were assessed. The results showed that the serum concentration of lipopolysaccharides (LPS) and tumor necrosis factor-alpha (TNF-α) in the colon of the animal consuming 0.5% and 2.0% of juçara pulp and the control group were statistically lower in the 0.5% group compared to the control group. On the other hand, the authors observed a significant decrease in the concentration of interleukin 1β in the groups fed with 0.5% and 2.0% compared to the control group. The TNF-α and the TNF-α/interleukin ratio was statistically lower in the group that consumed 0.5% of juçara lyophilized than the control group. The protein content of toll-like receptor-4 (TLR-4) was significantly lower in the rats’ groups fed with the diet supplemented with juçara compared to the control group. It can explain the decrease in the concentrations of proinflammatory cytokines. Moreover, an increase in interleukin in adipose tissue in the rats fed with juçara was reported by Argentato et al. [202], Morais et al. [203], and Freitas et al. [180].

Xie et al. [184] evaluated the effect of velutin, a flavonoid isolated from açaí fruit pulp, on decreasing TNF-α and interleukin-6 (IL-6) induced by lipopolysaccharide in peripheral macrophages and peritoneal macrophages of mice. The inhibition of the expression of TNF-α and IL-6 mRNA and protein levels in two macrophages was higher for velutin when compared to luteolin and apigenin. These flavonoids have been reported as the most effective in inhibiting the production of inflammatory cytokines [204,205].

The anti-inflammatory and antilipidemic activity of extracts rich in polyphenols (2.5–10 µg GAE mL^−1^) obtained from açaí was investigated using 3T3-L1 adipocytes [183]. The extracts inhibited the expression of mRNA and PPAR-γ protein and regulatory genes associated with lipid metabolisms such as aP2, FAS, FATP1, and LPL. In addition, açaí polyphenols with and without TNFα decreased the expression of proinflammatory cytokines, thus reducing the production of reactive oxygen species (ROS). The results were independent of the dose (2.5, 5, and 10 µg GAE mL^−1^). The bacaba phenolic extract showed the same behavior, demonstrating that BPE attenuates adipogenesis through downregulation of PPARγ2 and C/EBPα during differentiation’s early to middle stages. This, in turn, decreases the induction of metabolic genes associated with the adipocyte phenotype, such as FABP-4 and adiponectin [24].

The anti-inflammatory effects of defatted and lyophilized juçara pulp and its byproducts were evaluated in rats fed for four weeks with a high-fat diet [180]. The diet with supplementation of defatted and lyophilized juçara pulp was able to attenuate diet-induced nonalcoholic fatty liver disease (NAFLD). A decrease in inflammatory infiltrate, steatosis, and lipid peroxidation in the liver tissue was observed. These effects were correlated with the low lipid content and high content of polyphenolic compounds and anthocyanins in pulp of juçara.

Obesity is defined as a low-grade chronic inflammatory disease, and it can lead to several health problems, such as Type 2 diabetes, osteoarthritis, cancer, and cardiovascular disorders. Obesity is mainly caused by the positive energy balance resulting from a higher energy intake or a hypercaloric diet compared to the energy expenditure observed in a sedentary lifestyle [206,207,208]. The activation of inflammatory signaling pathways and atypical production of proinflammatory cytokines and fat storage cells (adipokines) are observed in chronic obesity [209,210]. It has been reported in the literature an antiobesity synergistic effect for phenolic compounds, flavonoids, carotenoids, and tocopherols [211,212]. The mechanism is related to the mediation of complex cell signaling pathways for lipolysis and β-oxidation of fatty acids, reducing the lipogenesis and adipogenesis [213].

Oyama et al. [214], Santamarina et al. [215], and Jamar et al. [216] reported a decrease in the inflammatory response in rats fed with a high-fat diet supplemented with juçara and its byproducts. Udani et al. [182] studied the influence of consuming 100 g of açaí pulp twice a day for one month in ten overweight adults. The results showed that the total cholesterol, LDL cholesterol, and the ratio of total cholesterol: HDL cholesterol, insulin, and plasma glucose decreased significantly compared to the control group. In addition, the consumption of açaí pulp for thirty days improved the postprandial rise in plasma glucose after the consumption of a standardized meal when compared to the placebo group.

### 5.4. Antitumoral/Antiproliferative Activity and Other Effects

The World Health Organization (WHO), in partnership with the International Agency for Research on Cancer (IARC), reported an increase in cancer from 18.1 million of cases in 2018 to 19 million in 2020, with 10 million deaths. It is expected that there will be 28.4 million new cases of cancer worldwide until the year 2040, which represents an increase of approximately 47% compared to 2020. In countries where the human development index (HDI) is considered low or medium, this expectation of cancer incidence for 2040 is estimated to increase by 96% when compared to 2020 [217].

Poor eating habit is considered the most important factor in the incidence of cancer and other noncommunicable diseases (NCDs) such as neurological, inflammatory, cardiovascular, and endocrine diseases [218]. Epidemiological studies show that a healthy lifestyle, which includes a balanced diet, can reduce the risk of several cancer types. A diet rich in fruits, vegetables, and grains, which are rich in phytochemicals, is widely recommended by several international bodies, such as the American Cancer Institute (AICR) and the World Cancer Research Foundation (WCRF) [219,220]. It has been reported that phenolic compounds promote the neoplastic effect and chemoprevention against cancer cells, reducing oxidative stress and modulating the signal transduction pathways involved in cell proliferation and survival.

Finco et al. [178] evaluated the in vitro antiproliferative potential of bacaba extracts and the apoptotic effect on the MCF-7 breast cancer cell line. Bacaba extract showed antiproliferative activity between 100–800 μg mL^−^^1^. Furthermore, a cell shrinkage and reduction of the cell monolayer area was observed in the MCF-7 breast cancer cells with the treatment at a concentration of 400 μg mL^−1^. The results indicated that the extracts obtained from bacaba induced apoptosis in MCF-7 cells. The authors suggested that bacaba extracts present chemopreventive potential and correlated such effects with dietary phenolics that are important preventive agents in cancer diseases.

Fuentes et al. [186] evaluated the effect of açaí oil nanoemulsion as a photosensitizer for photodynamic therapy (PPT) on the cell death of nonmetastatic melanoma in vitro and in vivo models. In vitro tests showed that the nanoemulsion induced the apoptosis of 85% of the melanoma cells and kept the normal cells viable. Moreover, the in vivo tests in mice showed that the photosensitizer formulated with açaí oil induced 82% reduction in the tumor of the animals submitted to the photodynamic therapy compared to the control group. The authors attributed the effect of anticancer to the polyphenols such as flavonoids, lignins, anthocyanins, and proanthocyanidins present in the açaí oil.

Boeing et al. [15] evaluated the antiproliferative effects of an ethanolic extract (80:20, *v*/*v*) obtained from the pulp and peel of butiá against cell lines strains of human intestinal cancer (Caco-2), cervical cancer lineage (HeLa), and human papillomavirus cells (SiHa and C33a). The ethanolic extract presented higher activity against the SiHa and C33a strains with a 50% inhibitory concentration (IC_50_) of 528 and 411 μg mL^−1^, respectively. On the other hand, the butiá extract did not reach half of the maximum inhibitory concentration (IC50) for the cell lines strains Caco-2 and HeLa. The treatment with the butiá extract did not affect the cell viability of murine fibroblasts and human keratinocytes. The chemopreventive activity was different between the cancer cell line strains. The variation in the chemopreventive activity among the cancer cells tested indicated different mechanisms of action for the ethanolic extract of butiá pulp. The authors indicated that (+)-catechin (259.00 mg kg^−1^), (−)-epicatechin (211.00 mg kg^−1^), and rutin (161 mg kg^−1^), present in the extract, were the compounds with bioactivity. The cytoprotective, antioxidant, cardioprotective, neuroprotective anticarcinogenic, and chemopreventive activities for these compounds are widely reported in the literature [221,222,223].

Medeiros et al. [191] investigated the effect of the consumption of buriti oil on somatic reflex development and retinol levels in neonatal rats. Thirty-six newborn male Wistar rats born to mothers who consumed a 7% lipid diet during pregnancy and lactation were used. The authors concluded that consumption of buriti oil interferes with weight gain and reflex maturation, accelerating tail growth and somatic development and increasing the availability of serum and hepatic retinol in newborn rats.

## 6. Conclusions

Arecaceae palm tree fruits have high nutritional value and are rich in bioactive compounds such as phenolic acids (gallic, vanillic, *p*-coumaric, and chlorogenic), flavonoids ((+) catechin, (−)-epicatechin, rutin, kaempferol), phytosterols, tocopherols, and carotenoids. These compounds are responsible for the antioxidant activity and potential health benefits such as antimicrobial, chemopreventive, cardioprotective, anti-inflammatory, and antiobesity effects. The Arecaceae palm tree fruits have potential for use in food, pharmaceutical, biotechnology, and cosmetic industries. Despite their rich nutritional and bioactive compounds composition, butiá (*Butia odorata*) and buritirana (*Mauritiella armata*) need more attention, considering the limited studies that have been reported in the literature. This review demonstrates the importance of valorizing underexploited Brazilian native fruits whose products and coproducts are rich in phytochemicals with potential benefits for human health. It also highlights the need for further research on the composition, health effects, processing, and full use of these raw materials. Moreover, the mechanisms and the synergism between the bioactive compounds of these fruits, and their effects, especially in human models, need to be further studied. In addition, government public policies and partnerships with local producers may improve the socioeconomic situation of extractivist populations, and can also encourage the consumption and processing of these fruits, ensuring the preservation of natural resources. This review can contribute to the dissemination of such products whose consumption and applications may help to promote a healthy diet.

## Figures and Tables

**Table 1:** Physicochemical characteristics and mineral composition of fruits belonging to the Arecaceae family.

Composition (g 100 g^−1^) (Fresh Weigh)	Bacaba (*O. bacaba)*	Patawa (*O. bataua)*	Juçara *(E. edulis)*	Açaí *(E. oleracea)*	Buriti *(M. flexuosa)*	Buritirana *(M. armata)*	Butiá (*B. odorata)*
Moisture	30.36	33.50	88.90	37.17	79.35	54.78	84.39
Ash	1.53	1.10	0.38	1.64	1.01	1.58	0.72
Lipids	21.02	14.40	4.36	8.06	7.72	21.01	2.18
Proteins	4.61	4.90	0.90	5.30	1.43	5.96	0.60
Total Fiber	-	29.70	27.10	-	6.02	65.46	1.31
* Carbohydrates	42.48	46.10	5.46	47.83	10.49	16.67	12.11
pH	5.83	-	4.47	5.23	4.05	-	3.17
** Total Acidity	0.22	-	0.48	1.20	0.47	-	2.17
Soluble Solids (°Brix)	-	-	3.03	6.46	4.33	-	15.50
Energy Value (kcal 100 g^−1^)	377.54	333.60	64.68	285.06	117.16	368.78	70.46
Minerals (mg 100 g^−1^)							
Calcium (Ca)	3.80	2.35	76.40	462.00	80.49	65.19	16.80
Magnesium (Mg)	7.80	41.23	47.4	317.00	40.34	49.12	12.50
Potassium (K)	173.35	2.17	419.10	930.00	218	672.65	462.4
Sodium (Na)	1.90	71.21	19.30	6.80	11.25	-	Trace
Phosphorus (P)	Trace	41.23	41.20	186.00	6.90	-	Trace
Nickel (Ni)	-	n.d.	1.00	-	0.06	-	Trace
Manganese (Mn)	0.67	0.61	3.10	45.00	1.79	3.55	0.03
Iron (Fe)	0.28	1.84	46.60	17.80	1.77	2.88	0.01
Zinc (Zn)	0.35	0.97	0.90	3.70	0.60	2.15	0.03
Cupper (Cu)	0.20	0.11	0.50	2.11	0.15	0.44	0.01
Selenium (Se)	-	Trace	0.50	Trace	0.05	-	-
Chromium (Cr)	-	-	-	-	0.12	-	Trace
References	[29,81]	[74,82]	[36,83,84]	[6,85,86,87]	[88,89,90]	[61]	[91,92,93]

* Carbohydrates calculated by difference [100 (moisture + ash + protein + lipid)]; ** total acidity expressed in mg citric acid 100 g^−1^; n.d.: not determined.

**Table 2:** Lipid composition of fruits belonging to the Arecaceae family.

Fatty Acids (%)	Bacaba (*O. bacaba)*	Patawa (*O. bataua)*	Juçara *(E. edulis)*	Açaí *(E. oleracea)*	Buriti *(M. flexuosa)*	Butiá (*B. odorata)*
Caproic (C6:0)	-	0.40	n.d.	n.d.	0.01	0.16
Caprylic (C8:0)	-	7.80	n.d.	n.d.	0.05	0.10
Capric (C10:0)	-	8.00	0.06	n.d.	0.01	0.08
Lauric (C12:0)	0.18	0.10	0.08	0.54	0.03	0.39
Myristic (C14:0)	0.59	0.09	0.05	0.65	0.12	1.60
Pentadecanoic (C15:0)	0.63	0.27	n.d.	0.07	0.07	n.d.
Palmitic (C16:0)	32.27	18.12	25.01	28.48	22.18	31.72
Margaric (C17:0)	n.d.	0.06	0.09	0.15	0.12	0.38
Stearic (C18:0)	4.70	1.74	3.51	4.46	2.51	4.43
Arachidic (C20:0)	0.48	0.07	0.26	0.08	0.11	0.79
Behenic (C22:0)	0.13	n.d.	0.08	-	0.02	1.57
Lignoceric (C24:0)	n.d.	n.d.	n.d.	n.d.	0.09	4.37
∑Saturated	38.98	36.65	29.14	30.23	27.76	45.59
Palmitoleic (C16:1 *cis* 9)	1.10	0.99	1.41	5.40	0.30	2.38
Oleic (C18:1 *cis* 9)	46.22	72.69	50.25	52.10	75.70	41.05
Gondoic (C20:1 *cis* 11)	n.d.	0.04	0.24	n.d.	0.58	0.46
∑Monounsaturated	47.32	73.72	51.90	57.50	76.58	43.89
Linoleic (C18:2 *cis* 9,12)	20.00	1.93	25.36	44.60	4,90	24.45
Linolenic (C18:3 *cis* 9,12,15)	1.93	0.79	0.74	4.39	8.20	8.35
∑Polyunsaturated	21.93	2.72	26.10	48.05	13.10	32.80
Tocopherols (mg kg^−1^)						
α-Tocopherol	148.41	56.50	571.00	645.00	614.00	-
β-Tocopherol	trace	7.80	472.00	-	761.87	-
γ-Tocopherol	trace	trace	150.00	-	56.71	-
δ-Tocopherol	-	7.70	trace	-	136.00	-
α-tocotrienol	-	n.d.	-	-	90.00	-
γ-tocotrienol	-	269.00	-	-	12.00	-
δ-tocotrienol	-	-	-	-	18.00	-
∑Tocopherols	148.41	341.00	1193.00	645.00	1688.58	-
Phytosterols (mg kg^−^^1^)						
β-Sitosterol + sitostanol	76.40	479.20	-	-	76.60	-
Campesterol	11.00	89.10	-	-	6.60	-
Campestanol	6.00	trace	-	-	-	-
Stigmasterol	12.60	166.10	-	-	16.80	-
Δ5-Avenasterol + Δ7-stigmasterol	trace	434.70	-	-	trace	-
Δ7-Avenasterol	-	-	-	-	-	-
Total	106.00	1169.10	-	-	100.00	-
References	[98,105,106]	[98,107,108]	[36,109]	[45,98,99,110,111]	[7,74,105,112,113,114,115]	[92]

n.d.: not determined.

**Table 3:** Essential amino acid in fruits belonging to the Arecaceae family and daily requirement of amino acids (DRAMA) for adults.

Essential Amino Acid (mg g^−1^ Protein)	DRAMA	Patawa (*O. bataua)*	Açaí *(E. oleracea)*	Buriti *(M. flexuosa)*
Isoleucine	30.00	47.00	3.96	14.20
Leucine	59.00	78.00	7.60	23.80
Lysine	45.00	53.00	6.45	19.00
Methionine	16.00	18.00	1.23	n.d.
Cystine	6.00	26.00	1.88	n.d.
Phenylalanine + Tyrosine	38	105.00	7.79	n.d.
Threonine	23.00	69.00	4.89	85.50
Valine	39.00	68.00	5.27	19.00
Tryptophan	6.00	9.00	1.54	23.80
Histidine	15.00	29.00	2.06	19.00
Total	277.00	502.00	42.67	204.30
References	[126]	[127]	[128,129]	[88]

n.d.: not determined.

**Table 4:** Bioactive compounds and antioxidant activity of extracts from fruits of the Arecaceae family.

Composition and Phenolics Profile (μg g^−1^)	Bacaba (*O. bacaba)*	Patawa (*O. bataua)*	Juçara *(E. edulis)*	Açaí *(E. oleracea)*	Buriti *(M. flexuosa)*	Butiá (*B. odorata)*
* Total phenolics	1759.27 ^b^	306.60 ^c^	5672.00 ^c^	3437.40 ^a^	435.08 ^c^	1250.30 ^b^
** Total anthocyanins	34.69 ^c^	68.04 ^b^	409.85 ^b^	110.10 ^c^	3.10 ^b^	25.13^b^
(+)-Catechin	20.21–3.85 ^c^	Trace ^c^	88.79 ^a^	Trace ^c^	961.21^b^	259.18 ^c^
(−)-Epicatechin	15.50–21.20 ^b^	8.70 ^c^	305.60 ^a^	Trace ^c^	1109.93 ^b^	211.12 ^c^
Quercetin	1.03–17.65 ^c^	0.68 ^c^	239.67 ^a^	135.66 ^c^	83.27 ^b^	360.19 ^b^
Myricetin	Trace ^b^	0.47 ^c^	660.00 ^a^	n.d.	145.11 ^b^	Trace ^b^
Apigenin	n.d.	0.05 ^c^	250.00 ^a^	12.57 ^c^	102.48 ^b^	0.09 ^c^
Luteolin	n.d.	0.03 ^c^	1020.00 ^a^	21.61 ^c^	1060.90 ^b^	0.44 ^c^
Kaempferol	n.d.	0.08 ^c^	440.00 ^a^	5.21 ^c^	41.54 ^b^	6.14 ^b^
*p*-Coumaric acid	Trace ^b^	0.50 ^c^	20.20 ^a^	3.08 ^c^	277.74 ^b^	0.77 ^c^
Caffeic acid	Trace ^b^	0.50 ^c^	3.80 ^c^	2.38 ^c^	895.53 ^b^	0.84 ^b^
Ferulic acid	4.77–10.80 ^b^	0.35 ^c^	46.00 ^c^	7.60 ^c^	184.66 ^b^	0.33 ^b^
Protocatechuic acid	n.d.	n.d.	66.02 ^a^	7.17 ^c^	2175.93 ^b^	Trace ^b^
Quinic acid	n.d.	Trace ^c^	Trace ^c^	n.d.	230.74 ^b^	Trace ^b^
Chlorogenic acid	0.71–64.56 ^c^	2.32 ^c^	16.50 ^a^	9.90 ^c^	1154.15 ^b^	290.10 ^b^
Gallic acid	40.45–1.26 ^c^	0.01 ^c^	7.50 ^c^	0.20 ^a^	0.06 ^c^	2.34 ^b^
Salicylic acid	n.d.	0.03 ^c^	2.66 ^a^	n.d.	0.16 ^c^	n.d.
Sinapic acid	2.15–9.72 ^b^	0.05 ^c^	29.90 ^c^	0.82 ^c^	0.34 ^c^	1.47 ^b^
Syringic acid	1.94–3.53 ^b^	0.70 ^c^	75.50 ^c^	19.03 ^c^	0.4 ^c^	Trace ^b^
Vanillic acid	Trace ^b^	0.98 ^c^	148.04 ^a^	46.55 ^c^	0.11 ^c^	0.07 ^b^
Naringenin	Trace ^b^	0.02 ^c^	5.49 ^a^	n.d.	Trace ^c^	0.24 ^c^
Isoquercitrin	n.d.	2.12 ^c^	24.77 ^a^	1.66 ^c^	5.85 ^c^	n.d.
Rutin	15.20–56.80 ^b^	0.65 ^c^	317.20 ^a^	34.07 ^c^	1460.00 ^b^	161.20 ^c^
Cyn 3-*O*-rutinoside	196.51–96.51 ^c^	470.00 ^c^	23.07 ^a^	1329.00 ^c^	n.d.	Trace ^b^
Antioxidant capacity						
DPPH (μmol TE g^−1^)	601 ^b^	2292.50 ^c^	724.92 ^c^	336.72 ^c^	1302.00 ^a^	64.70 ^c^
FRAP (μmol FeSO_4_ g^−1^)	65.67 ^b^	1869.90 ^c^	1745.33 ^a^	298.00 ^c^	8890.00 ^a^	-
ABTS (μmol TE g^−1^)	57.90 ^b^	2471.50 ^c^	64.50 ^b^	1154.43 ^c^	70.20 ^c^	-
ORAC (μmol TE g^−1^)	190.00 ^b^	1626.70 ^c^	1266.36 ^c^	1262.58 ^c^	2470.00 ^a^	278.15 ^c^
Carotenoids (mg kg^−1^)						
*Cis* lycopene	-	-	-	18.70 ^c^	n.d.	Trace ^b^
Lycopene	-	-	-	186.50 ^c^	n.d.	1.00 ^b^
*Cis* α-carotene	-	-	trace ^b^	n.d.	Trace ^b^	Trace ^b^
α-carotene	-	-	0.60 ^b^	n.d.	2.35 ^b^	Trace ^b^
*Cis* β-carotene	trace ^a^	-	trace ^b^	trace ^c^	Trace ^b^	10.20 ^b^
β-carotene	6.47 ^a^	-	86.12 ^b^	221.50 ^c^	52.57 ^b^	21.70 ^b^
Lutein	-	-	2.97 ^b^	483.00 ^c^	226.00 ^c^	4.70 ^b^
*Cis* lutein	-	-	0.13 ^b^	trace ^c^	Trace ^b^	Trace ^b^
Vitamins						
Vitamin A (RE 100 g^−1^)	-	n.d.	27.80 ^b^	300.60 ^a^	7280.00 ^b^	-
Vitamin C (mg 100 g^−1^)	30.20 ^b^	n.d.	186.00 ^b^	84.00 ^a^	59.93 ^b^	503.40 ^b^
Ascorbic Acid (mg 100 g^−1^)	0.90 ^b^	n.d.	n.d.	68.50 ^b^	51.85 ^b^	63.00 ^b^
References	[25,131,132,133,134]	[101,135,136]	[36,38,84,86,137,138,139,140,141]	[6,85,142,143,144,145,146,147,148,149]	[6,18,135,150,151,152,153,154]	[15,68,71,155,156]

^a^ Values expressed on a dry weight basis; ^b^ values expressed on a fresh weight basis; ^c^ freeze-dried sample. n.d.: not determined; trace: polyphenols identified by high-performance liquid chromatography below the limit of quantification; TE: trolox equivalents. * Total phenolics: mg gallic acid equivalent (GAE) 100 g^−1^; ** total anthocyanins: mg cyanidin 3-*O*-glucoside 100 g^−1^; ABTS: 2,2′ -azino-bis (3-ethylbenzothiazoline-6-sulphonic acid) free radical scavenging assay; DPPH: 2,2-diphenyl-1picrylhydrazyl free radical scavenging assay; ORAC: oxygen radical absorbance capacity assay; FRAP: ferric reducing antioxidant power; RAE: retinol activity equivalent.

**Table 5:** Effects on health for bioactive compounds extracted from fruits of the Arecaceae family.

Fruit	Source	Model	Health Effects	Sample Form	Effects	Related Compounds	References
Bacaba	Pulp extract	Cancer cells	Antiadipogenic effect	Lyophilized samples. Phenolic compounds were extracted with a mixture of acetone–water (80:20) (*v*/*v*). 140 g/600 mL of solvent/2 h of stirring.	↓ BPE: inhibits differentiation in 3T3-L1 preadipocytes. ↓ BPE: Downstabilizes protein expression of PPARγ2 and C/EBPα in a dose-dependent manner. ▪ It was checked that BPE attenuates adipogenesis through downregulation of PPARγ2 and C/EBPα during differentiation’s early to middle stages.	Phenolic compounds (gallic acid)	Lauvai et al. [24]
	Pulp extract	Cancer cells	Antiproliferative action on breast cancer cells	Lyophilized samples. Phenolic compounds were extracted with a mixture of acetone–water (80:20) (*v*/*v*). 20 g/400 mL of solvent/2 h of stirring.	↓ BPE: It acts in inhibiting cell proliferation mainly through the induction of apoptosis. ▪ The bacaba can be considered a fruit with chemopreventive potential. ▪ Regardless of the dose (*p* < 0.05), caspases -6, -8, and -9 were activated when correlated to untreated control.	Phenolic compounds (gallic acid) and caspase-activated deoxyribonuclease	Finco et al. [178]
	Pulp extract	In vivo and in vitro in cells	Antiproliferative effect	Lyophilized samples. The compounds of interest were extracted with acetone–water (80:20) (*v*/*v*) mixture. 20 g/400 mL of solvent/2 h of stirring.	↑ BPE demonstrated more significant antiproliferative activity than genipap extract, the target fruit of the same study. ↑ Antiproliferative capacity = IC_50_ of 649.6 ± 90.3 mg/mL in the MTT test and an IC_50_ of 1080.97 ± 0.7 mg/mL in the MUH. The MTT assay is more reliable when compared to other tests to assess the antiproliferative action.	Phenolic compounds	Finco et al. [179]
Patawa	Pulp oil	Insects	In vitro insecticidal activity	PPLM	Death of insect (*Sitophilus zeamais)* after 24 h.	Mono-, sesqui-, and diterpenes, limonoids and meliatoxins, including triterpenes, coumarins, and flavonoids	Santos et al. [132]
Juçara	Lyophilized pulp (LEE), the defatted lyophilized pulp (LDEE), and oil (EO)	Rats	Hypocholesterolemic effect in rats and antioxidant	Lyophilized samples (LEE). Oil extraction (18 g of LEE extracted with 600 mL of ethyl ether/12 h) (Soxhlet) (EO). The rest of the freeze-dried extract from the fruit was called LDEE.	↑ LEE is rich in polyunsaturated fatty acids. ↑ Right after degreasing, LEE and LDEE presented higher levels of anthocyanins and antioxidant capacity in vitro. ↓ The intake of LEE and LDEE, but not EO, attenuated diet-induced NAFLD. ↓ Reducing inflammatory infiltrate, steatosis, and lipid peroxidation in liver tissue. ▪ Only LDEE presented sufficient benefits to treat NAFLD in rats due to the high number of phenols and anthocyanins.	Phenols and anthocyanins	Freitas et al. [180]
	Juçara juice	Human	Control of fatigue, oxidative stress, and antioxidant	Not reported	JJ ↓ OSI immediately after an HIIT session. JJ ↑ GSH 1 h after an HIIT session. JJ ↑ total phenols and uric acid overtime during an HIIT session. JJ ↓ fatigue following an HIIT session.	Phenols, GSH, and uric acid	Copetti et al. [14]
	Juçara juice	Human	Antioxidant	PPLM	↑ JJ Ingestion promoted an increase in serum antioxidant capacity after one hour. ↑ Significant effects on GPx activity and FRAP results were observed. Interaction effect at time/treatment was observed on lipid peroxidation.	Phenolic compounds, anthocyanins, uric acid, and GSH	Cardoso et al. [171]
	Pulp	Rats	Antilipidemic and anti-inflammatory effects	Freeze-dried pulp for supplementation.	JS ↓ the proinflammatory cytokines in the colon. JS ↓ TLR-4 protein content in the colon. JS ↓ proinflammatory cytokines in EPI. ↓ TNF-α in EPI is independent of the LPS level.	Not specified	Silva et al. [181]
	Pulp	HT22 hippocampal cells	Neuroprotective	Lyophilized samples. The extracts were obtained with the following solvents: 1: hexane; 2: dichloromethane; 3: ethyl acetate; 4: butanol.	Dichloromethane extraction presented the ↑ levels of phenolics. Hexane extraction presented the ↓ levels of phenolics. ▪ Hexane and dichloromethane extracts exert a neuroprotective effect. ▪ HT22 neuronal cells were treated with crude extract and fractions of juçara fruits.	Phenolic compounds	Schulz et al. [141]
Açaí	Pulp	Human	Lipid-lowering effect	Pasteurized raw açaí pulp was safely consumed in this study at a dose of 100 g twice a day for one month.	Reduced fasting glucose, insulin, TC, LDL, and TC/HDL ratio and postprandial increase in plasma glucose.	Anthocyanins	Udani et al. [182]
	Concentrated and frozen juice	In vivo and in vitro tests in cell	PPLM	The compounds of interest were concentrated under vacuum using acidified (0.1% HCl) methanol and water. The methanol was evaporated in a rotary evaporator at <40 °C and redissolved in 60:40 (*v*/*v*) dimethyl sulphoxide (DMSO) and water, and stored at −80 °C.	↓ Expression of proinflammatory cytokines. ↓ Generation of reactive oxygen species. ↓ Cellular adhesion molecule. ↓ C/ebpα, C/ebpβ, Klf5, and Srebp1c.	Phenolic compounds (gallic acid), cyanidin-3-glucoside, and cyanidin-3-rutinoside	Martino et al. [183]
	Concentrate juice	In vivo and in vitro tests in cell	Antilipidemic and anti-inflammatory effects	Not related.	↓ Intracellular lipids by PPARƴ2. ↓ Adipogenic transcription factors, mRNA, proinflammatory cytokines. ↑ Adiponectin expression. ▪ The present study led to the discovery of a robust anti-inflammatory flavone, velutin.	Flavonoids, flavones, and velutin	Xie et al. [184]
	Pulp extract	In vivo and in vitro tests in cancer cell	Antitumor in vitro	Lyophilized samples. The compounds of interest were extracted with an ethanol–water (70:30) (*v*/*v*) mixture.	↑ Antitumoral effect against PCa DU145 cells involving downregulation of Bcl-2 gene. ▪ The synergism between açai and docetaxel is not so effective. ▪ The results suggest that açaí can be used as a dietary supplement to prevent PCa or disease progression.	Orientin and *p*-coumaric acid	Jobim et al. [185]
	Pulp oil	In vivo and in vitro tests in cancer cell	Anticancer	Data on obtaining açaí oil were not released. For the preparation of the nanoemulsion: 9 g of Tween 80^®^ surfactant; 2 g of açaí oil was mixed under stirring for 5 min at room temperature plus 25 mL of nanopure water, heated to 85 ° C. Then, 15 ml of water at 4 ° C was added. Concentration of 50 mg oil/mL.	↓ 82% reduction of the tumor when compared to control. ↓ Cell death occurred due to apoptosis/late necrosis. ▪ Important discoveries about the photodynamic properties of açaí oil = new photosensitizer.	Polyphenols (anthocyanin, proanthocyanidin, flavonoids, and lignans)	Fuentes et al. [186]
Butiá	Peel and pulp extract	In vitro tests	Antihyperglycemic and antioxidant	The compounds of interest were extracted with an ethanol–water (98:2) (*v*/*v*) mixture. 5 g/20 mL of solvent/5 min of stirring. Posteriorly, evaporated under pressure at 40 °C. Reconstituted with 20 ml of ethanol/water (3:1 (*v*/*v*)).	↓ Butiá extracts were not effective when compared to the control. ↑ Among the fruits used in the study, butiá extract was the most effective in reducing DPPH. ↓ Anthocyanins, phenolic compounds, reducing sugars, and carotenoids were responsible for the α-glucosidase inhibition.	Phenolic compounds (quercetin) and α-glucosidase	Vinholes et al. [187]
	Pulp extract	In vivo and in vitro tests in cancer cell	Antitumor and antioxidant	Lyophilized samples. The compounds of interest were extracted with 1 g and 10 ml of solvent in the following proportions: 1: methanol; 2: methanol: water (80:20, *v*/*v*); 3: ethyl acetate; 4: acetonitrile; 5: acetonitrile: water (80:20, *v*/*v*); 6: ethanol; 7: ethanol: water (80:20, *v*/*v*). At 30 ° C for 30 min, in an ultrasonic bath.	↑ Demonstrated antitumor activity against two cervical cancer cell lines, SiHa and C33a, evaluated by the MTT. ↑ High antioxidant activity. ↑ Positive correlation between the content of phenolic compounds and antitumor activity.	(+)-Catechin, (−)-epicatechin, and rutin	Boeing et al. [15]
	Pulp extract	In vivo and in vitro Deteriorating and pathogenic microorganisms	Antimicrobial	Lyophilized samples. The compounds of interest were extracted with methanol; 30 g/300 mL of solvent/2 h of stirring, then in an ultrasound bath (48 A/15 min).	↑ *Butiá odorata* extract showed high antimicrobial activity against the studied Salmonella strains. ↑ The zones of inhibition varied between 8 and 14 mm.	5-(hydroxymethyl)-2-furfural and piranone	Haubert et al. [188]
	Pulp extract	In vivo and in vitro Deteriorating and pathogenic microorganisms	Antimicrobial	Lyophilized samples. The compounds of interest were extracted with acetone; 30 g/300 mL of solvent/2 h of stirring (190 rpm).	↑ The extract of *Butiá odorata* showed antimicrobial activity against all strains of *E. coli*; ▪ The phytochemical profile of the extract showed as main compounds Z-10-pentadecenol (80.1%) and palmitic acid (19.4%). ↑ Antimicrobial activity against *E. coli* both in vitro and in situ.	Z-10-pentadecenol and palmitic acid	Maia et al. [189]
	Pulp extract	In vivo and in vitro Deteriorating and pathogenic microorganisms	Antimicrobial	Lyophilized samples. The compounds of interest were extracted with methanol or hexane; 30 g/300 mL of methanol or hexane/2 h of stirring, then in an ultrasound bath (48 A/15 min). BHE: Butiá odorata hexane extract. BME: Butiá odorata methanol extract.	↑ BHE and BME: antibacterial activity against all tested pathogenic bacteria (*S. aureus*, *L. monocytogenes*, *B. cereus*, *S. Typhimurium, E. coli*, and *P. aeruginosa*). ↑ BHE and BME ↓. ↑ BHE: contained γ-sitosterol as a significant component. ▪ Good alternative to synthetic preservatives to increase shelf life and food safety.	γ-sitosterol	Maia et al. [190]
Buriti	Pulp oil	In vitro tests	Antioxidant and antimicrobial	The extracts were obtained from 800 g of fruit pulp during 6 to 8 h of extractions with the following reagents: chloroform (FCB), ethyl acetate (FAB), and ethanol (FEB) (Soxhlet). Antioxidant analysis by the ABTS and FRAP method.	↑↓ (FCB), (FAB), and (FEB) = moderate antioxidant activity. ↑ Antimicrobial activity, antibiotic-enhancing. ↑ High potential in the development of therapeutic alternatives against resistant bacteria. ↓ Failed to modulate antifungal activity.	Phenolic compounds (catechin, caffeic acid, rutin, orientin, luteolin, and others) and flavones; flavanol; flavanonols; catechins	Nonato et al. [18]
	Pulp, shell, and endocarp	Rats	In vitro and ex vivo chemopreventive action	Samples lyophilized. The compounds of interest were extracted with methanol (1:10; sample/solvent); 1 g/10 mL of solvent/48 h (stored at 4 °C).	↑ The antioxidant analysis of the parts of *M. flexuosa* showed promising chemopreventive potential. ↑ More significant results were found for the bark. ▪ None of the extracts induced lysis of rat erythrocytes, being able to protect blood cells.	Phenol, flavonoid, condensed tannin	Freire et al. [177]
	Pulp and bark oil	In vivo and in vitro Deteriorating and pathogenic microorganisms	Antimicrobial	PPLM	↓ Crude oil has low antimicrobial activity. ↑ Nanoencapsulated oil showed a great increase in antimicrobial activity. ↑ Emulsion technique: increased the antimicrobial activity of buriti oil by 59, 62, and 43% against *Pseudomonas aeruginosa, Klebsiella pneumonia*, and *Staphylococcus aureus*, respectively. ↑ Plant-based products are more efficient for Gram-positive bacteria than Gram-negative bacteria.	Quercetin, eugenol, vanillin and tannins, ellagic acid, and catechin	Castro et al. [16]
	Pulp oil	In vivo and in vitro test in cancer cell	Hydroxypterocarpans with estrogenic activity	Dry pulp. The powdered pulp was defatted three times with n-hexane (2 L) at 40 °C, after which the residue was extracted with ethanol (3 L) for 2 hours at 60 °C. Finally, an ethanol extract (124.8 g) (Soxhlet).	▪ Lespeflorin G8 was identified as a significant estrogenic compound. ▪ Was found to be a receptor estrogen agonist. ▪ 8-HHP was a partial agonist bound to ER. ▪ First study to have found estrogenic compounds in the buriti oil fraction.	Two hydroxypterocarpans = lespeflorin G8 (LF), 8-hydroxy-homo pterocarpan (8-HHP); and 17β-Estradiol	Shimoda et al. [19]
	Crude and refined oil	Rats	Hypocholesterolemic effect in rats	The compounds of interest were extracted with chloroform–methanol (2:1) (*v*/*v*); 1 g/20 mL of solvent/3 min.	↓ Total cholesterol. ↓ LDL. ↓ Triglycerides. ↓ AST. Maternal consumption of buriti oil ↓ weight gain and reflex maturation, but ↑ somatic maturation in newborn rats. ↑ Increases the deposition of serum retinol and liver in the offspring.	Serum retinol and liver retinol	Medeiros et al. [191]

**↓**: Decreased. **↑**: Increased. PPLM: product purchased on the local market, without specifications of how it was prepared; BPE: bacaba phenolic extract; MTT [3-(4,5-dimethylthiazol-2-yl)-2,5-diphenyltetrazolium bromide]; MUH (4-methylumbelliferyl heptanoate); LF: lespeflorin G8; 8-HHP: 8-hydroxy-homo pterocarpan; ER: estrogen receptor; AST: aspartate aminotransferase; PPARƴ2: peroxisome proliferator-activated receptor-gamma; C/ebpα, C/ebpβ, Klf5, and Srebp1 c: adipogenic transcription factors; LDL: low-density lipoprotein; HDL: high-density lipoprotein; AST: aspartate aminotransferase; PCa: prostate cancer; MTT: 3-(4,5-dimethyl-2-thiazolyl)-2,5-diphenyl-2H-tetrazolium bromide; SiHa and C33a: human cancer cell; NAFLD: nonalcoholic fatty liver disease; JJ: juçara juice; HIIT: high-intensity interval training; GSH: glutathione; OSI: oxidative stress index; FRAP: ferric reducing antioxidant power; GPx: glutathione peroxidase; JS: juçara supplementation; EPI: epididymal adipose tissue; LPS: lipopolysaccharide; TNF-α: tumor necrosis factor-alpha; TLR-4: toll-like receptor-4.

## Data Availability

Not applicable.

## References

[1] Valli M., Russo H.M., Bolzani V.S. (2018). The potential contribution of natural products from Brazilian biodiversity to the bioeconomy. Ann. Braz. Acad. Sci..

[2] Santos D.C.D., Oliveira Filho J.G.D., Sousa T.L.D., Ribeiro C.B., Egea M.B. (2021). Ameliorating effects of metabolic syndrome with the consumption of rich-bioactive compounds fruits from Brazilian Cerrado: A narrative review. Crit. Rev. Food Sci. Nutr..

[3] GBIF Global Biodiversity Information Facility Secretariat. https://www.gbif.org/species/7681.

[4] Teixeira G.L., Ibañez E., Block J.M. (2022). Emerging Lipids from Arecaceae Palm Fruits in Brazil. Molecules.

[5] Santos O.V., Langley A.C.D.C.P., Lima A.J.M., Moraes V.S.V., Soares S.D., Teixeira-Costa B.E. (2022). Nutraceutical potential of Amazonian oilseeds in modulating the immune system against COVID-19–A narrative review. J. Funct. Foods.

[6] Souza F.G., Araújo F.F., Paulo Farias D., Zanotto A.W., Neri-Numa I.A., Pastore G.M. (2020). Brazilian fruits of Arecaceae family: An overview of some representatives with promising food, therapeutic and industrial applications. Food Res. Int..

[7] Speranza P., Alves J., Macedo A. (2016). Amazonian Buriti oil: Chemical characterization and antioxidant potential. Grasas Y Aceites.

[8] Cardoso A.L., Liz S., Rieger D.K., Farah A.C.A., Vieira F.G.K., Assis M.A.A., Di Pietro P.F. (2018). An update on the biological activities of *Euterpe edulis* (Juçara). Planta Med..

[9] Corrêa B.M., Baldissera L., Barbosa F.R., Ribeiro E.B., Andrighetti C.R., Silva Agostini J., Sousa Valladao D.M. (2019). Centesimal and mineral composition and antioxidant activity of the bacaba fruit peel. Biosci. J..

[10] Hoek V.D.Y., Solas S.Á., Peñuela M.C. (2019). The palm *Mauritia flexuosa*, a keystone plant resource on multiple fronts. Biodivers. Conserv..

[11] Barbosa J.R., Júnior R.N.C. (2022). Food sustainability trends-How to value the açaí production chain for the development of food inputs from its main bioactive ingredients?. Trends Food Sci. Technol..

[12] CONAB (2020). Companhia Nacional de Abastecimento.

[13] Guimarães L.A.O.P., Souza R.G. (2017). Palmeira Juçara: Patrimônio Natural da Mata Atlântica no Espírito Santo.

[14] Copetti C.L.K., Orssatto L.B., Diefenthaeler F., Silveira T.T., Silva E.L., Liz S., Di Pietro P.F. (2020). Acute effect of juçara juice (*Euterpe edulis* Martius) on oxidative stress biomarkers and fatigue in a high-intensity interval training session: A single-blind cross-over randomized study. J. Funct. Foods.

[15] Boeing J.S., Barizão É.O., Rotta E.M., Volpato H., Nakamura C.V., Maldaner L., Visentainer J.V. (2020). Phenolic compounds from *Butia odorata* (Barb. Rodr.) noblick fruit and its antioxidant and antitumor activities. Food Anal. Methods.

[16] Castro G.M.M.A., Passos T.S., Cruz Nascimento S.S., Medeiros I., Araújo N.K., Maciel B.L.L., Assis C.F. (2020). Gelatin nanoparticles enable water dispersibility and potentialize the antimicrobial activity of Buriti (*Mauritia flexuosa*) oil. BMC Biotechnol..

[17] Lakmal K., Yasawardene P., Jayarajah U., Seneviratne S.L. (2021). Nutritional and medicinal properties of Star fruit (*Averrhoa carambola*): A review. Food Sci. Nutr..

[18] Nonato C.D.F.A., Leite D.O.D., Pereira R.C., Boligon A.A., Ribeiro-Filho J., Rodrigues F.F.G., Costa J.G.M. (2018). Chemical analysis and evaluation of antioxidant and antimicrobial activities of fruit fractions of *Mauritia flexuosa* L. f. (Arecaceae). PeerJ.

[19] Shimoda H., Takeda S., Takarada T., Kato Y., Shimizu N., Toda K., Matsuda H. (2019). Hydroxypterocarpans with estrogenic activity in Aguaje, the fruit of *Mauritia flexuosa* (*Peruvian moriche* palm). Bioact. Compd. Health Dis..

[20] Teixeira N., Melo J.C., Batista L.F., Paula-Souza J., Fronza P., Brandao M.G. (2019). Edible fruits from Brazilian biodiversity: A review on their sensorial characteristics versus bioactivity as tool to select research. Food Res. Int..

[21] Araujo F.F., Neri-Numa I.A., Paulo Farias D., Cunha G.R.M.C., Pastore G.M. (2019). Wild Brazilian species of *Eugenia genera* (Myrtaceae) as an innovation hotspot for food and pharmacological purposes. Food Res. Int..

[22] Lopes E., Soares-Filho B., Souza F., Rajão R., Merry F., Ribeiro S.C. (2019). Mapping the socio-ecology of Non-Timber Forest Products (NTFP) extraction in the Brazilian Amazon: The case of açaí (*Euterpe precatoria* Mart) in Acre. Landsc. Urban Plan..

[23] Cavalcante P.B. (2010). Frutas Comestíveis na Amazonia.

[24] Lauvai J., Schumacher M., Finco F.D.B.A., Graeve L. (2017). Bacaba phenolic extract attenuates adipogenesis by down-regulating PPARγ and C/EBPα in 3T3-L1 cells. NFS J..

[25] Finco F.A. (2012). Health Enhancing Traditional Foods in Brazil: An Interdisciplinary Approach to Food and Nutritional Security.

[26] Cól C.D., Tischer B., Flôres S.H., Rech R. (2021). Foam-mat drying of bacaba (*Oenocarpus bacaba*): Process characterization, physicochemical properties, and antioxidant activity. Food Bioprod. Process..

[27] Nascimento R.A.D., Andrade E.L., Santana E.B., Ribeiro N.F.D.P., Costa C.M.L., Faria L.J.G.D. (2019). Bacaba powder produced in spouted bed: An alternative source of bioactive compounds and energy food product. Braz. J. Food Technol..

[28] Jesus C.R.D., Oliveira M.N.D., Souza Filho M.F.D., Silva R.A.D., Zucchi R.A. (2008). First record of *Anastrepha parishi* Stone (*Diptera, Tephritidae*) and its host in Brazil. Rev. Bras. De Entomol..

[29] Seixas F.R.F., Sesquim E.A.R., Raasch G.S., Cíntra D.E. (2016). Característica físico-química e perfil lipídico da bacaba proveniente da Amazônia ocidental. Braz. J. Food Res..

[30] Seixas F.R.F., Vieira T.S., Cintra D.E.C. (2016). Caracterização físico-química e da fração lipídica do patauá proveniente da aldeia baixa verde no município de Alto Alegre dos Parecis-RO. Rev. Científica Da Unesc..

[31] Henderson A., Galeano G., Bernal R. (2019). Field Guide to the Palms of the Americas.

[32] Hidalgo P.S., Rita de Cássia S.N., Nunomura S.M. (2016). Plantas oleaginosas amazônicas: Química e atividade antioxidante de patauá (*Oenocarpus bataua* Mart.). Rev. Virtual De Química.

[33] Rogez H. (2000). Açaí: Preparo, Composição e Melhoramento da Conservação.

[34] Schulz M., Borges G.D.S.C., Gonzaga L.V., Costa A.C.O., Fett R. (2016). Juçara fruit (*Euterpe edulis* Mart.): Sustainable exploitation of a source of bioactive compounds. Food Res. Int..

[35] Lorenzi H., Noblick L., Kahn F., Ferreira E. (2010). Flora Brasileira—Arecaceae (Palmeiras).

[36] Borges G.D.S.C., Vieira F.G.K., Copetti C., Gonzaga L.V., Zambiazi R.C., Mancini Filho J., Fett R. (2011). Chemical characterization, bioactive compounds, and antioxidant capacity of jussara (*Euterpe edulis*) fruit from the Atlantic Forest in southern Brazil. Food Res. Int..

[37] Bicudo M.O.P., Ribani R.H. (2015). Anthocyanins and Antioxidant Properties of Juçara Fruits (*Euterpe edulis* M.) Along the On-tree Ripening Process. Blucher Biochem. Proc..

[38] Bicudo M.O.P., Ribani R.H., Beta T. (2014). Anthocyanins, phenolic acids and antioxidant properties of juçara fruits (*Euterpe edulis M*.) along the on-tree ripening process. Plant Foods Hum. Nutr..

[39] Schirmann G., Reis T., Goudel F., Miller P.R.M., Silva E., Block J.M. (2013). Frutos da palmeira-juçara: Alimento de qualidade para os catarinenses. Agropecuária Catarin.

[40] Schulz M., Seraglio S.K.T., Brugnerotto P., Gonzaga L.V., Costa A.C.O., Fett R. (2020). Composition and potential health effects of dark-colored underutilized Brazilian fruits–A review. Food Res. Int..

[41] Yamaguchi K.K.L., Pereira L.F.R., Lamarão C.V., Lima E.S., Veiga-Junior V.F. (2015). Amazon açaí: Chemistry and biological activities: A review. Food Chem..

[42] Strudwick J., Sobel G. (1988). Uses of Euterpe oleracea Mart. in the Amazon Estuary, Brazil. Adv. Econ. Bot..

[43] Torma P.D.C.M.R., Brasil A.V.S., Carvalho A.V., Jablonski A., Rabelo T.K., Moreira J.C.F., Oliveira Rios A. (2017). Hydroethanolic extracts from different genotypes of açaí (*Euterpe oleracea*) presented antioxidant potential and protected human neuron-like cells (SH-SY5Y). Food Chem..

[44] Silva M.P., Cunha V.M.B., Sousa S.H.B., Menezes E.G.O., Bezerra P.N., Neto J.T.F., Junior R.N.C. (2019). Supercritical CO_2_ extraction of lyophilized Açaí (*Euterpe oleracea* Mart.) pulp oil from three municipalities in the state of Pará, Brazil. J. CO2 Util..

[45] Bichara C.M.G., Rogez H. (2011). Açai (*Euterpe oleracea* Martius). Postharvest Biology and Technology of Tropical and Subtropical Fruits.

[46] Oliveira M.D.S.P., Neto J.T.F., Pena R.S. (2007). Açaí: Técnicas de cultivo e processamento. CEP.

[47] Jesus A.L.T., Cristianini M., Santos N.M., Júnior M.R.M. (2020). Effects of high hydrostatic pressure on the microbial inactivation and extraction of bioactive compounds from açaí (*Euterpe oleracea* Martius) pulp. Food Res. Int..

[48] Jesus A.L.T., Leite T.S., Cristianini M. (2018). High isostatic pressure and thermal processing of açaí fruit (*Euterpe oleracea* Martius): Effect on pulp color and inactivation of peroxidase and polyphenol oxidase. Food Res. Int..

[49] Oliveira D.N., Claro P.I., Freitas R.R., Martins M.A., Souza T.M., Silva B.M., Bufalino L. (2019). Enhancement of the Amazonian Açaí Waste Fibers through Variations of Alkali Pretreatment Parameters. Chem. Biodivers..

[50] IBGE (2021). Tabela 5457—Área Plantada ou Destinada à Colheita, área Colhida, Quantidade Produzida, Rendimento Médio e Valor da Produção das Lavouras Temporárias e Permanentes. In Sist. IBGE Recuper. https://sidra.ibge.gov.br/tabela/5457.

[51] Reis A.F., Schmiele M. (2019). Características e potencialidades dos frutos do Cerrado na indústria de alimentos. Braz. J. Food Technol..

[52] Silva R.S., Ribeiro L.M., Mercadante-Simões M.O., Nunes Y.R.F., Lopes P.S.N. (2014). Seed structure and germination in buriti (*Mauritia flexuosa*), the Swamp palm. Flora-Morphol. Distrib. Funct. Ecol. Plants.

[53] Carneiro T.B., Mello Carneiro J.G. (2011). Frutos e polpa desidratada buriti, *Mauritia flexuosa* L.: Aspectos físicos, químicos e tecnológicos. Rev. Verde De Agroecol. E Desenvolv. Sustentável.

[54] Vieira I.R., Oliveira J.S., Santos K.P., Silva G.O., Vieira F.J., Barros R.F. (2016). A contingent valuation study of buriti (*Mauritia flexuosa* Lf) in the main region of production in Brazil: Is environmental conservation a collective responsibility?. Acta Bot. Bras..

[55] Martins R.C., Filgueiras T.D.S., Albuquerque U.P. (2014). Use and diversity of palm (Arecaceae) resources in central western Brazil. Sci. World J..

[56] Sandri D.D.O., Xisto A.L.R.P., Rodrigues E.C., Morais E.C.D., Barros W.M.D. (2017). Antioxidant activity and physicochemical characteristics of buriti pulp (*Mauritia flexuosa*) collected in the city of Diamantino–MTS. Rev. Bras. De Frutic..

[57] Manhães L., Menezes E., Marques A., Sabaa Srur A. (2015). Flavored buriti oil (*Mauritia flexuosa*, Mart.) for culinary usage: Innovation, production and nutrition value. J. Culin. Sci. Technol..

[58] Koolen H.H., Silva F.M., Silva V.S., Paz W.H., Bataglion G.A. (2018). Buriti fruit—*Mauritia flexuosa*. Exotic Fruits.

[59] Smith N. (2014). Palms and People in the Amazon.

[60] Anunciação P.C., Giuffrida D., Murador D.C., Filho G.X.P., Dugo G., Pinheiro-Sant’Ana H.M. (2019). Identification and quantification of the native carotenoid composition in fruits from the Brazilian Amazon by HPLC–DAD–APCI/MS. J. Food Compos. Anal..

[61] Souza F.G., Araújo F.F., Orlando E.A., Rodrigues F.M., Chávez D.W.H., Pallone J.A.L., Pastore G.M. (2022). Characterization of Buritirana (*Mauritiella armata*) Fruits from the Brazilian Cerrado: Biometric and Physicochemical Attributes, Chemical Composition and Antioxidant and Antibacterial Potential. Foods.

[62] Reitz R. (1988). Flora ilustrada catarinense. Plantas Fascic Calyceraceas.

[63] Geymonat G., Rocha N. (2009). M´botia, Ecosistema Único en el Mundo.

[64] Moura R.C.D., Lopes P.S.N., Junior D.D.S.B., Gomes J.G., Pereira M.B. (2010). Fruit and seed biometry of *Butia odorata* (Mart.) *Beccari* (Arecaceae), in the natural vegetation of the North of Minas Gerais, Brazil. Biota Neotrop..

[65] Rivas M., Jaurena M., Gutiérrez L., Barbieri R.L. (2014). Diversidade vegetal do campo natural de *Butia odorata* (Barb. Rodr.) Noblick no Uruguai. Agrociencia Urug..

[66] Marchi M.M., Barbieri R.L., Sallés J.M., Costa F.A.D. (2018). Flora herbácea e subarbustiva associada a um ecossistema de butiazal no Bioma Pampa. Rodriguésia.

[67] Lorenzi H., Souza H.M., Costa J.T.M., Cerqueira L.S.C., Ferreira E. (2004). Palmeiras brasileiras e exóticas cultivadas. Nova Odessa Inst. Plant..

[68] Má C., Dunshea F.R., Suleria H.A. (2019). Lc-esi-qtof/ms characterization of phenolic compounds in palm fruits (jelly and *fishtail palm*) and their potential antioxidant activities. Antioxidants.

[69] Rosa L., Castellani T.T., Reis A. (1998). Reproductive biology of *Butia capitata* (Martius) Beccari var. *odorata* in coastal sandy shrub vegetation in Laguna, SC. Rev. Bras. De Botânica.

[70] Schwartz E., Fachinello J.C., Barbieri R.L., Silva J.B.D. (2010). Avaliação de populações de Butia capitata de Santa Vitória do Palmar. Revista Brasileira de Fruticultura. Rev. Bras. De Frutic..

[71] Hoffmann J.F., Zandoná G.P., Santos P.S., Dallmann C.M., Madruga F.B., Rombaldi C.V., Chaves F.C. (2017). Stability of bioactive compounds in butiá *(Butia odorata)* fruit pulp and nectar. Food Chem..

[72] Garcia-Amezquita L.E., Tejada-Ortigoza V., Heredia-Olea E., Serna-Saldívar S.O., Welti-Chanes J. (2018). Differences in the dietary fiber content of fruits and their by-products quantified by conventional and integrated AOAC official methodologies. J. Food Compos. Anal..

[73] Stephen A.M., Champ M.M.J., Cloran S.J., Fleith M., Lieshout L.V., Mejborn H., Burley V.J. (2017). Dietary fibre in Europe: Current state of knowledge on definitions, sources, recommendations, intakes and relationships to health. Nutr. Res. Rev..

[74] Darnet S.H., Silva L.H.M.D., Rodrigues A.M.D.C., Lins R.T. (2011). Nutritional composition, fatty acid and tocopherol contents of buriti (*Mauritia flexuosa*) and patawa (*Oenocarpus bataua*) fruit pulp from the Amazon region. Food Sci. Technol..

[75] Simpson H.L., Campbell B.J. (2015). Dietary fibre—Microbiota interactions. Aliment. Pharmacol. Ther..

[76] Damasceno N.R.T., Vila A.S., Cofán M., Heras A.M.P., Fitó M., Gutiérrez V.R., Ros E. (2013). Mediterranean diet supplemented with nuts reduces waist circumference and shifts lipoprotein subfractions to a less atherogenic pattern in subjects at high cardiovascular risk. Atherosclerosis.

[77] White P.J., Broadley M.R. (2009). Biofortification of crops with seven mineral elements often lacking in human diets–iron, zinc, copper, calcium, magnesium, selenium and iodine. New Phytol..

[78] WHO (2012). World Health Organization. Guideline: Potassium Intake for Adults and Children.

[79] Sobotka L., Allison S., Stanga Z. (2008). Basics in clinical nutrition: Water and electrolytes in health and disease. e-SPEN Eur. E-J. Clin. Nutr. Metab..

[80] Ballesta M.C.M., Perles R.D., Moreno D.A., Muries B., López C.A., Bastías E., Carvajal M. (2010). Minerals in plant food: Effect of agricultural practices and role in human health. A review. Agron. Sustain. Dev..

[81] Aguiar J.P.L., Amaral Souza F.D.C. (2018). Bacaba (*Oenocarpus bacaba*): A new wet tropics nutritional source. Afr. J. Agric. Res..

[82] Saravia S.A.M., Montero I.F., Linhares B.M., Santos R.A., Marcia J.A.F. (2020). Mineralogical Composition and Bioactive Molecules in the Pulp and Seed of Patauá (*Oenocarpus bataua* Mart.): A Palm from the Amazon. Int. J. Plant Soil Sci..

[83] Ribeiro L.O., Mendes M.F., Pereira C.D.S.S. (2011). Avaliação da composição centesimal, mineral e teor de antocianinas da polpa de juçaí (*Euterpe edulis* Martius). Rev. Eletrônica TECCEN.

[84] Inada K.O.P., Oliveira A.A., Revorêdo T.B., Martins A.B.N., Lacerda E.C.Q., Freire A.S., Monteiro M.C. (2015). Screening of the chemical composition and occurring antioxidants in jabuticaba (*Myrciaria jaboticaba*) and jussara (*Euterpe edulis*) fruits and their fractions. J. Funct. Foods.

[85] Gordon A., Cruz A.P.G., Cabral L.M.C., Freitas S.C., Taxi C.M.A.D., Donangelo C.M., Marx F. (2012). Chemical characterization and evaluation of antioxidant properties of Açaí fruits (*Euterpe oleraceae* Mart.) during ripening. Food Chem..

[86] Silva N.A., Rodrigues E., Mercadante A.Z., Rosso V.V. (2014). Phenolic Compounds and Carotenoids from Four Fruits Native from the Brazilian Atlantic Forest. J. Agric. Food Chem..

[87] Ferreira D.S., Gomes A.L., Silva M.G., Alves A.B., Angol W.H.D., Ferrari R.A., Pacheco M.T.B. (2016). Antioxidant capacity and chemical characterization of açaí (*Euterpe oleracea* Mart.) fruit fractions. Food Sci. Technol..

[88] Manhães L.R.T., Sabaa-Srur A.U.O. (2011). Centesimal composition and bioactive compounds in fruits of buriti collected in Pará. Food Sci. Technol..

[89] Cândido T.L.N., Silva M.R. (2017). Comparison of the physicochemical profiles of buriti from the Brazilian Cerrado and the Amazon region. Food Sci. Technol..

[90] Schiassi M.C.E.V., Souza V.R., Lago A.M.T., Campos L.G., Queiroz F. (2018). Fruits from the Brazilian Cerrado region: Physico-chemical characterization, bioactive compounds, antioxidant activities, and sensory evaluation. Food Chem..

[91] Faria J.P., Almeida F., Silva L.C.R.D., Vieira R.F., Agostini-Costa T.D.S. (2008). Caracterização da polpa do coquinho-azedo (Butia). Rev. Bras. De Frutic..

[92] Ferrão T.S., Ferreira D.F., Flores D.W., Bernardi G., Link D., Barin J.S., Wagner R. (2013). Evaluation of composition and quality parameters of jelly palm *(Butia odorata)* fruits from different regions of Southern Brazil. Food Res. Int..

[93] Pereira M.C., Boschetti W., Rampazzo R., Celso P.G., Hertz P.F., Rios A.D.O., Flores S.H. (2014). Mineral characterization of native fruits from the southern region of Brazil. Food Sci. Technol..

[94] Bergman C., Gray-Scott D., Chen J.J., Meacham S. (2009). What is next for the dietary reference intakes for bone metabolism related nutrients beyond calcium: Phosphorus, magnesium, vitamin D, and fluoride?. Crit. Rev. Food Sci. Nutr..

[95] Shenkin A. (2008). Basics in clinical nutrition: Physiological function and deficiency states of trace elements. e-SPEN Eur. E J. Clin. Nutr. Metab..

[96] Thomas V.L., Gropper S.S. (1996). Effect of chromium nicotinic acid supplementation on selected cardiovascular disease risk factors. Biol. Trace Elem. Res..

[97] Combs G.F. (2004). Status of selenium in prostate cancer prevention. Br. J. Cancer.

[98] Montúfar R., Laffargue A., Pintaud J.C., Hamon S., Avallone S., Dussert S. (2010). *Oenocarpus bataua* Mart. (Arecaceae): Rediscovering a source of high oleic vegetable oil from Amazonia. J. Am. Oil Chem. Soc..

[99] Rufino M.S.M., Pérez-Jiménez J., Arranz S., Alves R.E., Brito E.S., Oliveira M.S., Saura-Calixto F. (2011). Açaí (*Euterpe oleraceae*) ‘BRS Pará’: A tropical fruit source of antioxidant dietary fiber and high antioxidant capacity oil. Food Res. Int..

[100] Portinho J.A., Zimmermann L.M., Bruck M.R. (2012). Efeitos benéficos do açaí. Int. J. Nutrol..

[101] Rezaire A., Robinson J.C., Bereau D., Verbaere A., Sommerer N., Khan M.K., Fils-Lycaon B. (2014). Amazonian palm *Oenocarpus bataua* (“patawa”): Chemical and biological antioxidant activity—Phytochemical composition. Food Chem..

[102] Oliveira R.M.M., Pereira F.T., Pereira E.C., Mendonça C.J.S. (2020). Óleo de buriti: Índice de qualidade nutricional e efeito antioxidante e antidiabético. Rev. Virtual De Quím..

[103] Hauss D.J. (2007). Oral lipid-based formulations. Adv. Drug Deliv. Rev..

[104] Feeney O.M., Crum M.F., McEvoy C.L., Trevaskis N.L., Williams H.D., Pouton C.W., Porter C.J. (2016). 50 years of oral lipid-based formulations: Provenance, progress and future perspectives. Adv. Drug Deliv. Rev..

[105] Santos M.F.G., Alves R.E., Méndez M.V.R. (2013). Minor components in oils obtained from Amazonian palm fruits. Grasas Y Aceites.

[106] Santos M.F.G., Marmesat S., Brito E.S., Alves R.E., Dobarganes M.C. (2013). Major components in oils obtained from Amazonian palm fruits. Grasas Y Aceites.

[107] Hernandez N.P.B., Fregapane G., Moya M.D.S. (2009). Bioactive compounds, volatiles and antioxidant activity of virgin Seje oils (*Jessenia bataua*) from the Amazonas. J. Food Lipids.

[108] Mushtaq M., Akram S., Hasany S.M. (2019). Seje (*Oenocarpus/Jessenia bataua*) Palm Oil. Fruit Oils: Chemistry and Functionality.

[109] Carvalho A.G., Silva K.A., Silva L.O., Costa A.M., Akil E., Coelho M.A., Torres A.G. (2019). Jussara berry (*Euterpe edulis M*.) oil-in-water emulsions are highly stable: The role of natural antioxidants in the fruit oil. J. Sci. Food Agric..

[110] Duncan C.E. (2010). Factors Influencing the Stability and Marketability of a Novel, Phytochemical-Rich Oil from the Açai Palm Fruit (*Euterpe oleracea* Mart.). Ph.D. Thesis.

[111] Santos D.D.S., Klauck V., Campigotto G., Alba D.F., Reis J.H., Gebert R.R., Silva A.S. (2019). Benefits of the inclusion of açai oil in the diet of dairy sheep in heat stress on health and milk production and quality. J. Therm. Biol..

[112] Silva S.M., Sampaio K.A., Taham T., Rocco S.A., Ceriani R., Meirelles A.J. (2009). Characterization of oil extracted from buriti fruit (*Mauritia flexuosa*) grown in the Brazilian Amazon region. J. Am. Oil Chem. Soc..

[113] Serra J.L., Rodrigues A.M.C., Freitas R.A., Meirelles A.J.A., Darnet S.H., Silva L.H.M. (2019). Alternative sources of oils and fats from Amazonian plants: Fatty acids, methyl tocols, total carotenoids and chemical composition. Food Res. Int..

[114] Cruz M.B., Oliveira W.S., Araújo R.L., França A.C.H., Pertuzatti P.B. (2020). Buriti (Mauritia flexuosa L.) pulp oil as an immunomodulator against enteropathogenic Escherichia coli. Ind. Crops Prod..

[115] Mesquita J.D.A., Oliveira T.T.D.S., Santos J.G.D.S.D., Gaspar M.R.G.R.D.C., Vieira V.D.A., Rodrigues E.C., Faria R.A.P.G.D. (2020). Fatty acid profile and physicochemical characterization of buriti oil during storage. Ciência Rural.

[116] Swiglo A.G., Sikorska E., Khmelinskii I., Sikorski M. (2007). Tocopherol content in edible plant oils. Pol. J. Food Nutr. Sci..

[117] Firestone D. (2013). Physical and Chemical Characteristics of Oils, Fats, and Waxes.

[118] Azzi A. (2019). Tocopherols, tocotrienols and tocomonoenols: Many similar molecules but only one vitamin E. Redox Biol..

[119] Ghaedi E., Foshati S., Ziaei R., Beigrezaei S., Varkaneh H.K., Ghavami A., Miraghajani M. (2020). Effects of phytosterols supplementation on blood pressure: A systematic review and meta-analysis. Clin. Nutr..

[120] Ribas S.A., Sichieri R., Moreira A.S.B., Souza D.O., Cabral C.T.F., Gianinni D.T., Cunha D.B. (2017). Phytosterol-enriched milk lowers LDL-cholesterol levels in Brazilian children and adolescents: Double-blind, cross-over trial. Nutr. Metab. Cardiovasc. Dis..

[121] Dumolt J.H., Rideout T.C. (2017). The lipid-lowering effects and associated mechanisms of dietary phytosterol supplementation. Curr. Pharm. Des..

[122] Jones P.J., Shamloo M., MacKay D.S., Rideout T.C., Myrie S.B., Plat J., Weingärtner O. (2018). Progress and perspectives in plant sterol and plant stanol research. Nutr. Rev..

[123] Zhang S.B., Lu Q.Y., Yang H., Li Y., Wang S. (2011). Aqueous enzymatic extraction of oil and protein hydrolysates from roasted peanut seeds. JAOCS J. Am. Oil Chem. Soc..

[124] Grohmann U., Bronte V. (2010). Control of immune response by amino acid metabolism. Immunol. Rev..

[125] Lindseth G., Helland B., Caspers J. (2015). The effects of dietary tryptophan on affective disorders. Arch. Psychiatr. Nurs..

[126] WHO (2007). Protein and Amino Acid Requirements in Human Nutrition.

[127] Balick M.J., Gershoff S.N. (1981). Nutritional evaluation of the Jessenia bataua palm: Source of high-quality protein and oil from tropical America. Econ. Bot..

[128] Schauss A.G. (2010). Acai (*Euterpe oleracea* Mart.): A macro and nutrient rich palm fruit from the Amazon rain forest with demonstrated bioactivities in vitro and in vivo. Bioactive Foods in Promoting Health.

[129] McCusker S., Buff P.R., Yu Z., Fascetti A.J. (2014). Amino acid content of selected plant, algae and insect species: A search for alternative protein sources for use in pet foods. J. Nutr. Sci..

[130] Royo A.V., Rocha J.A., Santos K.T., Freitas J.F.L., Almeida C.A., Ribeiro B., Júnior A.F.M. (2019). Comparative Studies Between *Mauritia flexuosa* and *Mauritiella Armata*. Pharmacogn. J..

[131] Canuto G.A.B., Xavier A.A.O., Neves L.C., Benassi M.D.T. (2010). Caracterização físico-química de polpas de frutos da Amazônia e sua correlação com a atividade anti-radical livre. Rev. Bras. De Frutic..

[132] Santos A.C.V., Fernandes C.C., Lopes L.M., Sousa A.H. (2015). Use of plant oils from the southwestern Amazon for the control of maize weevil. J. Stored Prod. Res..

[133] Carvalho A.V., Silveira T.F., Sousa S.H.B., Moraes M.R., Godoy H.T. (2016). Phenolic composition and antioxidant capacity of bacaba genotypes. J. Food Compos. Anal..

[134] Sousa S.H.B., Mattietto R.A., Chisté R.C., Carvalho A.V. (2018). Phenolic compounds are highly correlated to the antioxidant capacity of genotypes of *Oenocarpus bacaba* Mart. fruits. Food Res. Int..

[135] Tauchen J., Bortl L., Huml L., Miksatkova P., Doskocil I., Marsik P., Kokoska L. (2016). Phenolic composition, antioxidant and anti-proliferative activities of edible and medicinal plants from the Peruvian Amazon. Rev. Bras. De Farmacogn..

[136] Costa T.S.A. (2018). Bioactive compounds and health benefits of some palm species traditionally used in Africa and the Americas—A review. J. Ethnopharmacol..

[137] Rufino M.S.M., Alves R.E., Brito E.S., Jiménez J.P., Calixto F.S., Filho J.M. (2010). Bioactive compounds and antioxidant capacities of 18 non-traditional tropical fruits from Brazil. Food Chem..

[138] Carvalho A.G.S., Machado M.T.C., Silva V.M., Sartoratto A., Rodrigues R.A.F., Hubinger M.D. (2016). Physical properties and morphology of spray dried microparticles containing anthocyanins of jussara (*Euterpe edulis* Martius) extract. Powder Technol..

[139] Peron D.V., Fraga S., Antelo F. (2017). Thermal degradation kinetics of anthocyanins extracted from juçara (*Euterpe edulis* Martius) and “Italia” grapes (*Vitis vinifera* L.), and the effect of heating on the antioxidant capacity. Food Chem..

[140] Vieira G.S., Marques A.S., Machado M.T., Silva V.M., Hubinger M.D. (2017). Determination of anthocyanins and non-anthocyanin polyphenols by ultra-performance liquid chromatography/electrospray ionization mass spectrometry (UPLC/ESI–MS) in jussara (*Euterpe edulis*) extracts. J. Food Sci. Technol..

[141] Schulz M., Gonzaga L.V., Souza V., Farina M., Vitali L., Micke G.A., Fett R. (2019). Neuroprotective effect of juçara (*Euterpe edulis* Martius) fruits extracts against glutamate-induced oxytosis in HT22 hippocampal cells. Food Res. Int..

[142] Agawa S., Sakakibara H., Iwata R., Shimoi K., Hergesheimer A., Kumazawa S. (2011). Anthocyanins in mesocarp/epicarp and endocarp of fresh açai (*Euterpe oleracea Mart.)* and their antioxidant activities and bioavailability. Food Sci. Technol. Res..

[143] Kang J., Thakali K.M., Xie C., Kondo M., Tong Y., Ou B., Wu X. (2012). Bioactivities of açaí (*Euterpe precatoria* Mart.) fruit pulp, superior antioxidant and anti-inflammatory properties to Euterpe oleracea Mart. Food Chem..

[144] Bataglion G.A., Silva F.M., Eberlin M.N., Koolen H.H. (2015). Determination of the phenolic composition from Brazilian tropical fruits by UHPLC—MS/MS. Food Chem..

[145] Neves L.T.B.C., Campos D.C.D.S., Mendes J.K.S., Urnhani C.O., Araújo K.G. (2015). Qualidade de frutos processados artesanalmente de açaí (*Euterpe oleracea* Mart.) e bacaba (*Oenocarpus bacaba* Mart.). Rev. Bras. De Frutic..

[146] Carvalho A.V., Silveira T.F., Mattietto R.D.A., Oliveira M.D.S.P., Godoy H.T. (2017). Chemical composition and antioxidant capacity of açaí (*Euterpe oleracea*) genotypes and commercial pulps. J. Sci. Food Agric..

[147] Garzón G.A., Cuenca C.E.N., Vincken J.P., Gruppen H. (2017). Polyphenolic composition and antioxidant activity of açai (*Euterpe oleracea Mart*.) from Colombia. Food Chem..

[148] Fragoso M.F., Romualdo G.R., Vanderveer L.A., Barraza J.F., Cukierman E., Clapper M.L., Barbisan L.F. (2018). Lyophilized açaí pulp (*Euterpe oleracea Mart*) attenuates colitis-associated colon carcinogenesis while its main anthocyanin has the potential to affect the motility of colon cancer cells. Food Chem. Toxicol..

[149] Minighin E.C., Souza K.F., Valenzuela V.D.C.T., Silva N.D.O.C., Anastácio L.R., Labanca R.A. (2019). Effect of in vitro gastrointestinal digestion on the mineral content, phenolic compounds, and antioxidant capacity of commercial pulps of purple and white açaí *(Euterpe oleracea* Mart.). J. Food Sci. Technol..

[150] Rosso V.V., Mercadante A.Z. (2007). Identification and quantification of carotenoids, by HPLC-PDA-MS/MS, from Amazonian fruits. J. Agric. Food Chem..

[151] Costa P.A., Ballus C.A., Filho J.T., Godoy H.T. (2010). Phytosterols and tocopherols content of pulps and nuts of Brazilian fruits. Food Res. Int..

[152] Bataglion G.A., Silva F.M., Eberlin M.N., Koolen H.H. (2014). Simultaneous quantification of phenolic compounds in buriti fruit (*Mauritia flexuosa* Lf) by ultra-high performance liquid chromatography coupled to tandem mass spectrometry. Food Res. Int..

[153] Cândido T.L.N., Silva M.R., Agostini-Costa T.S. (2015). Bioactive compounds and antioxidant capacity of buriti (*Mauritia flexuosa* Lf) from the Cerrado and Amazon biomes. Food Chem..

[154] Hamacek F.R., Lucia C.M.D., Silva B.P.D., Moreira A.V.B., Sant’Ana H.M.P. (2018). Buriti of the cerrado of Minas Gerais, Brazil: Physical and chemical characterization and content of carotenoids and vitamins. Food Sci. Technol..

[155] Beskow G.T., Hoffmann J.F., Teixeira A.M., Fachinello J.C., Chaves F.C., Rombaldi C.V. (2015). Bioactive and yield potential of jelly palms (Butia Odorata Barb. Rodr.). Food Chem..

[156] Hoffmann J.F., Barbieri R.L., Rombaldi C.V., Chaves F.C. (2014). Butia spp. (Arecaceae): An overview. Sci. Hortic..

[157] Romualdo G.R., Fragoso M.F., Borguini R.G., Santiago M.C.P.A., Fernandes A.A.H., Barbisan L.F. (2015). Protective effects of spray-dried açaí (*Euterpe oleracea* Mart) fruit pulp against initiation step of colon carcinogenesis. Food Res. Int..

[158] Del Pozo-Insfran D., Brenes C.H., Talcott S.T. (2004). Phytochemical composition and pigment stability of Açai (*Euterpe oleracea* Mart.). J. Agric. Food Chem..

[159] Al-Rejaie S.S., Aleisa A.M., Sayed-Ahmed M.M., Hafez M.M. (2013). Protective effect of rutin on the antioxidant genes expression in hypercholestrolemic male Westar rat. BMC Complement. Altern. Med..

[160] Paula C.S., Canteli V.C.D., Hirota B.C.K., Campos R., Oliveira V.B., Kalegari M., Miguel M.D. (2014). Potencial antioxidante in vitro das folhas da *Bauhinia ungulata* L. Rev. De Ciências Farm. Básica E Apl..

[161] Rodriguez-Amaya D.B. (1996). Assessment of the provitamin A contents of foods: The Brazilian experience. J. Food Compos. Anal..

[162] Vuong L., Franke A., Custer L., Murphy S. (2006). Momordica cochinchinensis Spreng. (gac) carotenoids de fruits reevaluates. J. Food Compos. Anal..

[163] Lima A.L.D.S., Lima K.D.S.C., Coelho M.J., Silva J.M., Godoy R.L.D.O., Pacheco S. (2009). Avaliação dos efeitos da radiação gama nos teores de carotenoides, ácido ascórbico e açúcares do futo buriti do brejo (*Mauritia flexuosa* L.). Acta Amaz..

[164] Hamano P.S., Mercadante A.Z. (2001). Composition of carotenoids from commercial products of caja (*Spondias lutea*). J. Food Compos. Anal..

[165] Mercadante A.Z., Rodriguez-Amaya D.B. (1998). Effects of ripening, cultivar differences, and processing on the carotenoid composition of mango. J. Agric. Food Chem..

[166] Niizu P.Y., Rodriguez-Amaya D.B. (2005). Flowers and leaves of *Tropaeolum majus* L. as rich sources of lutein. J. Food Sci..

[167] Caba Z.T. (2019). The concept of superfoods in diet. In Role Altern. Innov. Food Ingred. Prod. Consum. Wellness.

[168] Mditshwa A., Magwaza L.S., Tesfay S.Z., Opara U.L. (2017). Postharvest factors affecting vitamin C content of citrus fruits: A review. Sci. Hortic..

[169] Couto M.A.L., Canniatti-Brazaca S.G. (2010). Quantification of vitamin C and antioxidant capacity of citrus varieties. Food Sci. Technol..

[170] Berman A.Y., Motechin R.A., Wiesenfeld M.Y., Holz M.K. (2017). The therapeutic potential of resveratrol: A review of clinical trials. NPJ Precis. Oncol..

[171] Cardoso A.L., Pietro P.F., Vieira F.G.K., Boaventura B.C.B., Liz S., Borges G.D.S.C., Silva E.L. (2015). Acute consumption of juçara juice (*Euterpe edulis*) and antioxidant activity in healthy individuals. J. Funct. Foods.

[172] Umeno A., Biju V., Yoshida Y. (2017). In vivo ROS production and use of oxidative stress-derived biomarkers to detect the onset of diseases such as Alzheimer’s disease, Parkinson’s disease, and diabetes. Free Radic. Res..

[173] Dudonne S., Vitrac X., Coutiere P., Woillez M., Mérillon J.M. (2009). Comparative study of antioxidant properties and total phenolic content of 30 plant extracts of industrial interest using DPPH, ABTS, FRAP, SOD, and ORAC assays. J. Agric. Food Chem..

[174] Leba L.J., Brunschwig C., Saout M., Martial K., Bereau D., Robinson J.C. (2016). *Oenocarpus bacaba* and *Oenocarpus bataua* leaflets and roots: A new source of antioxidant compounds. Int. J. Mol. Sci..

[175] Hidalgo G.I., Almajano M.P. (2017). Red fruits: Extraction of antioxidants, phenolic content, and radical scavenging determination: A review. Antioxidants.

[176] Nicácio A.E., Rotta E.M., Boeing J.S., Barizão É.O., Kimura E., Visentainer J.V., Maldaner L. (2017). Antioxidant activity and determination of phenolic compounds from Eugenia involucrata DC. Fruits by UHPLC-MS/MS. Food Anal. Methods.

[177] Freire J.A.P., Oliveira G.L.D.S., Lima L.K.F., Ramos C.L.S., Medeiros S.R.A., Lima A.C.S.D., Ferreira P.M.P. (2018). In vitro and ex vivo chemopreventive action of *Mauritia flexuosa* products. Evid.-Based Complement. Altern. Med..

[178] Finco F.D.B.A., Kloss L., Graeve L. (2016). Bacaba (*Oenocarpus bacaba*) phenolic extract induces apoptosis in the MCF-7 breast cancer cell line via the mitochondria-dependent pathway. NFS J..

[179] Finco F.D.A., Böser S., Graeve L. (2013). Antiproliferative activity of Bacaba (*Oenocarpus bacaba*) and Jenipapo (*Genipa americana* L.) phenolic extracts: A comparison of assays. Nutr. Food Sci..

[180] Freitas R.B., Novaes R.D., Gonçalves R.V., Mendonça B.G., Santos E.C., Ribeiro A.Q., Leite J.P.V. (2016). *Euterpe edulis* extract but not oil enhances antioxidant defenses and protects against nonalcoholic fatty liver disease induced by a high-fat diet in rats. Oxid. Med. Cell. Longev..

[181] Silva F.P., Miranda D.A., Carnier M., Maza P.K., Boldarine V.T., Rischiteli A.S., Oyama L.M. (2021). Low dose of Juçara pulp (*Euterpe edulis* Mart.) minimizes the colon inflammatory milieu promoted by hypercaloric and hyperlipidic diet in mice. J. Funct. Foods.

[182] Udani J.K., Singh B.B., Singh V.J., Barrett M.L. (2011). Effects of Acai (*Euterpe oleracea* Mart.) berry preparation on metabolic parameters in a healthy overweight population: A pilot study. Nutr. J..

[183] Martino H.S.D., Dias M.M.S., Noratto G., Talcott S., Talcott S.U.M. (2016). Anti-lipidaemic and anti-inflammatory effect of açai (*Euterpe oleracea* Martius) polyphenols on 3T3-L1 adipocytes. J. Funct. Foods.

[184] Xie C., Kang J., Li Z., Schauss A.G., Badger T.M., Nagarajan S., Wu X. (2012). The açaí flavonoid velutin is a potent anti-inflammatory agent: Blockade of LPS-mediated TNF-α and IL-6 production through inhibiting NF-κB activation and MAPK pathway. J. Nutr. Biochem..

[185] Jobim M.L., Barbisan F., Fortuna M., Teixeira C.F., Boligon A.A., Ribeiro E.E., Cruz I.B.M. (2019). Açai (*Euterpe oleracea,* Mart.), an Amazonian fruit has antitumor effects on prostate cancer cells. Arch. Biosci. Health.

[186] Fuentes M.V., Muehlmann L.A., Longo J.P.F., Silva J.R., Fascineli M.L., Souza P., Azevedo R.B. (2017). Photodynamic therapy mediated by acai oil (*Euterpe oleracea* Martius) in nanoemulsion: A potential treatment for melanoma. J. Photochem. Photobiol. B Biol..

[187] Vinholes J., Lemos G., Barbieri R.L., Franzon R.C., Vizzotto M. (2017). In vitro assessment of the antihyperglycemic and antioxidant properties of araçá, butiá and pitanga. Food Biosci..

[188] Haubert L., Zehetmeyr M.L., Pereira Y.M.N., Kroning I.S., Maia D.S.V., Sehn C.P., Silva W.P. (2019). Tolerance to benzalkonium chloride and antimicrobial activity of *Butia odorata Barb*. *Rodr*. extract in Salmonella spp. isolates from food and food environments. Food Res. Int..

[189] Maia D.S.V., Haubert L., Soares K.S., Würfel S.D.F.R., Silva W.P. (2019). *Butia odorata Barb. Rodr.* extract inhibiting the growth of *Escherichia coli* in sliced mozzarella cheese. J. Food Sci. Technol..

[190] Maia D., Aranha B., Chaves F., Silva W. (2017). Antibacterial activity of *Butiá odorata* extracts against pathogenic bacteria. Trends Phytochem. Res..

[191] Medeiros M.C., Aquino J.S., Soares J., Figueiroa E.B., Mesquita H.M., Pessoa D.C., Stamford T.M. (2015). Buriti oil (*Mauritia flexuosa* L.) negatively impacts somatic growth and reflex maturation and increases retinol deposition in young rats. Int. J. Dev. Neurosci..

[192] Shan B., Cai Y.Z., Brooks J.D., Corke H. (2007). The in vitro antibacterial activity of dietary spice and medicinal herb extracts. Int. J. Food Microbiol..

[193] Tortora G.J., Funke B.R., Case C.L. (2012). Microbiologia.

[194] Polovková M., Šimko P. (2017). Determination and occurrence of 5-hydroxymethyl-2-furaldehyde in white and brown sugar by high performance liquid chromatography. Food Control.

[195] Leão K.M.M., Reis L.V.C., Speranza P., Rodrigues A.P., Ribeiro A.P.B., Macedo J.A., Macedo G.A. (2019). Physicochemical characterization and antimicrobial activity in novel systems containing buriti oil and structured lipids nanoemulsions. Biotechnol. Rep..

[196] Maleki S.J., Crespo J.F., Cabanillas B. (2019). Anti-inflammatory effects of flavonoids. Food Chem..

[197] Baliga M.S., Baliga B.R.V., Kandathil S.M., Bhat H.P., Vayalil P.K. (2011). A review of the chemistry and pharmacology of the date fruits (*Phoenix dactylifera* L.). Food Res. Int..

[198] Pérez A.D.L., López N.L., Grijalva E.P.G., Heredia J.B. (2016). Phenolic compounds: Natural alternative in inflammation treatment. A Review. Cogent Food Agric..

[199] Zhang L., Virgous C., Si H. (2019). Synergistic anti-inflammatory effects and mechanisms of combined phytochemicals. J. Nutr. Biochem..

[200] Sharma S., Naura A.S. (2020). Potential of phytochemicals as immune-regulatory compounds in atopic diseases: A review. Biochem. Pharmacol..

[201] Alarcon De La Lastra C., Villegas I. (2005). Resveratrol as an anti-inflammatory and anti-aging agent: Mechanisms and clinical implications. Mol. Nutr. Food Res..

[202] Argentato P.P., Morais C.A., Santamarina A.B., de Cássia César H., Estadella D., de Rosso V.V., Pisani L.P. (2017). Jussara (*Euterpe edulis* Mart.) supplementation during pregnancy and lactation modulates UCP-1 and inflammation biomarkers induced by trans-fatty acids in the brown adipose tissue of offspring. Clin. Nutr. Exp..

[203] Morais A.C., Oyama L.M., Oliveira J.L.D., Garcia M.C., Rosso V.V.D., Amigo L.S.M., Pisani L.P. (2014). Jussara (*Euterpe edulis* Mart.) supplementation during pregnancy and lactation modulates the gene and protein expression of inflammation biomarkers induced by trans-fatty acids in the colon of offspring. Mediat. Inflamm..

[204] Hougee S., Sanders A., Faber J., Graus Y.M., Van den Berg W.B., Garssen J., Hoijer M.A. (2005). Decreased pro-inflammatory cytokine production by LPS-stimulated PBMC upon in vitro incubation with the flavonoids apigenin, luteolin or chrysin, due to selective elimination of monocytes/macrophages. Biochem. Pharmacol..

[205] Shanmugam K., Holmquist L., Steele M., Stuchbury G., Berbaum K., Schulz O., Münch G. (2008). Plant-derived polyphenols attenuate lipopolysaccharide-induced nitric oxide and tumour necrosis factor production in murine microglia and macrophages. Mol. Nutr. Food Res..

[206] Lykouras L., Michopoulos J. (2011). Transtornos de ansiedade e obesidade. Psychiatriki.

[207] Pervanidou P., Chrousos G.P. (2011). Stress and obesity/metabolic syndrome in childhood and adolescence. Int. J. Pediatr. Obes..

[208] Schrempf J. (2014). A social connection approach to corporate responsibility: The case of the fast-food industry and obesity. Bus. Soc..

[209] Solinas G. (2012). Molecular pathways linking metabolic inflammation and thermogenesis. Obes. Rev..

[210] Byers T., Sedjo R.L. (2015). Body fatness as a cause of cancer: Epidemiologic clues to biologic mechanisms. Endocr. Relat. Cancer.

[211] Nuutila A.M., Puupponen-Pimiä R., Aarni M., Oksman-Caldentey K.M. (2013). Comparison of antioxidant activities of onion and garlic extracts by inhibition of lipid peroxidation and radical scavenging activity. Food Chem..

[212] Singh J., Upadhyay A.K., Prasad K., Bahadur A., Rai M. (2007). Variability of carotenes, vitamin C, E and phenolics in Brassica vegetables. J. Food Compos. Anal..

[213] Wang S., Moussa N.M., Chen L., Mo H., Shastri A., Su R., Shen C.L. (2014). Novel insights of dietary polyphenols and obesity. J. Nutr. Biochem..

[214] Oyama L.M., Silva F.P.D., Carnier J., Miranda D.A., Santamarina A.B., Ribeiro E.B., Rosso V.V. (2016). Juçara pulp supplementation improves glucose tolerance in mice. Diabetol. Metab. Syndr..

[215] Santamarina A.B., Jamar G., Mennitti L.V., Ribeiro D.A., Cardoso C.M., Rosso V.V., Pisani L.P. (2019). Polyphenols-rich fruit (*Euterpe edulis* Mart.) prevents peripheral inflammatory pathway activation by the short-term high-fat diet. Molecules.

[216] Jamar G., Santamarina A.B., Mennitti L.V., Cesar H.C., Oyama L.M., Rosso V.V., Pisani L.P. (2018). *Bifidobacterium* spp. reshaping in the gut microbiota by low dose of juçara supplementation and hypothalamic insulin resistance in Wistar rats. J. Funct. Foods.

[217] IARC (2020). All Cancers-International Agency for Research on Cancer. https://www.iarc.who.int/faq/latest-global-cancer-data-2020-qa/.

[218] Marmot M., Atinmo T., Byers T., Chen J., Hirohata T., Jackson A., James W., Kolonel L., Kumanyika S., Leitzmann C. (2007). Food, Nutrition, Physical Activity, and the Prevention of Cancer: A Global Perspective.

[219] Kushi L.H., Doyle C., McCullough M., Rock C.L., Wahnefried W.D., Bandera E.V., American Cancer Society Nutrition and Physical Activity Guidelines Advisory Committee (2012). American Cancer Society Guidelines on nutrition and physical activity for cancer prevention: Reducing the risk of cancer with healthy food choices and physical activity. CA A Cancer J. Clin..

[220] Perera P.S., Thompson R.L., Wiseman M.J. (2012). Recent evidence for colorectal cancer prevention through healthy food, nutrition, and physical activity: Implications for recommendations. Curr. Nutr. Rep..

[221] Castro A.J.A., Domínguez F., Carrancá A.G. (2013). Rutin exerts antitumor effects on nude mice bearing SW480 tumor. Arch. Med. Res..

[222] Costa R., Silva L., Kinsey A.L. (2014). Health benefits of nongallated and gallated flavan-3-ols: A prospectus. Recent Advances in Gallate Research.

[223] Ganeshpurkar A., Saluja A.K. (2017). The pharmacological potential of rutin. Saudi Pharm. J..

